# Polymer Casting and Water Immersion-Based Large-Area Graphene Transfer for Flexible Electronics Fabrication

**DOI:** 10.1021/acsami.5c23601

**Published:** 2026-02-16

**Authors:** Andrea Zuccaro, Ekin G. Simsar, Naomi Addai Asante, Tugce Dogruel, Lan Wang, Tejasvini Malakalapalli, Piran R. Kidambi, Hasan Erbil Abaci, Margot Damaser, Metin Uz

**Affiliations:** † Chemical and Biomedical Engineering, 2564 Cleveland State University, 2121 Euclid Avenue, Cleveland, Ohio 44115, United States; ‡ Biomedical Engineering, Lerner Research Institute, 2569 Cleveland Clinic, 9620 Carnegie Avenue N Building, Cleveland, Ohio 44106, United States; § Mechanical and Aerospace Engineering, 3463 University of Florida, 939 Center Drive, Gainesville, Florida 32611, United States; ∥ Dermatology and Biomedical Engineering, 5798 Columbia University, 622 West 168th Street, New York, New York 10032, United States; ⊥ Advanced Platform Technology Center, Louis Stokes Cleveland VA Medical Center, 10701 East Boulevard, Cleveland, Ohio 44106, United States; # Glickman Urological and Kidney Institute, Cleveland Clinic, 2050 East 96th St Q Building, Cleveland, Ohio 44106, United States

**Keywords:** large-area graphene transfer, biodegradable and implantable devices, polymer casting, water immersion, cytocompatibility

## Abstract

This study focuses on developing an efficient large-area graphene transfer method that combines high-throughput and precise laser engraving, simple polymer casting, and water immersion to fabricate conductive graphene and biodegradable polymer-based implantable flexible electronic devices. The low-temperature treatment of graphene sheets on a glass substrate reduced graphene sheet roughness and increased hydrophobicity, enabling facile and high-efficiency (∼100%) large-area graphene transfer to a flexible polymer substrate. This method also benefited from differences in the work of adhesion at the graphene sheet/glass substrate and the graphene sheet/flexible polymer substrate interfaces. The transferred graphene sheets showed stability, structural integrity, and high conductivity (∼40 Ω/sq sheet resistance) under in vitro and in vivo mimicking conditions. The low-temperature-treated and laser-engraved conductive graphene patterns, transferred on a flexible and biodegradable polymer substrate, demonstrated in vitro cytocompatibility on different cells. Two flexible electronic devices (1—a graphene coil-integrated electrode cuff and 2—an interdigitated graphene cuff-integrated piezoelectric device) were fabricated using the developed method, and both demonstrated functionality and proof of concept by generating output voltages that can enhance cell/tissue regeneration. In addition, the ease of handling, ex vivo implantation, and feasibility of suturing were demonstrated by performing implantation surgeries on the pudendal nerve in cadaveric rats. Overall, this promising large-area graphene transfer method can be used to fabricate biodegradable, implantable devices that can serve as interfaces to stimulate cells and tissues for regeneration and repair.

## Graphical abstract

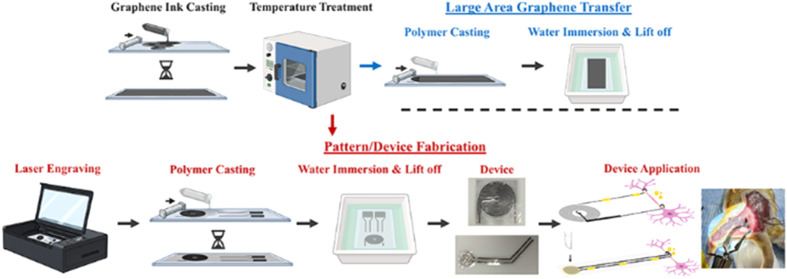

## 1 Introduction

The field of flexible electronics has been gaining interest to develop efficient and convenient devices, particularly for different biomedical applications, including, but not limited to, healthcare monitoring, wearable electronics, sensors, and implantable devices.^1^ Conventionally available flexible electronics fabrication processes often require harsh chemical and physical treatments to transfer the conductive material to the flexible substrate. In general, the materials used during these processes should maintain the structural integrity as well as the flexibility without losing function.^2^ One such material, graphene, has been drawing attention in the field of flexible electronics for its electrical, optical, and mechanical properties.^3^

Fabrication of graphene-based flexible electronics conventionally employs chemical vapor deposition (CVD), which allows cost-effective large-scale production of graphene films on a metal substrate at high temperatures (>200 up to >1000 °C).^4–6^ After CVD-grown conductive graphene is obtained on a metal substrate, it is transferred onto the final substrate of interest. This intermediate transfer step is generally done by polymer-based transfer or thermal release tape, which relies on the surface energy and adhesion forces between polymer (or tape)/graphene and polymer (or tape)/target substrate interfaces.^7–9^ Recent studies demonstrated water-based large-area graphene transfer (A4-size graphene sheets) through intercalation of water between the graphene and metal substrate (e.g., copper). This water-based transfer mechanism occurs through the galvanic coupling between graphene and the metal substrate surface, resulting in accelerated oxidation/corrosion and subsequent peeling of graphene from the surface using a polymer support layer.^10–14^ Although promisingly advancing the field, these transfer methods still suffer from certain challenges: (1) substantial post-processing (e.g., stamping, chemical/physical etching, washing, high temperature annealing (>200 up to >1000 °C), high pressure or separation from the intermediate substrate), (2) maintaining the integrity and structure of transferred graphene, and (3) remaining residues affecting the electrical properties and cytocompatibility of the final material.^4,7–9^

As an alternative to CVD-grown graphene, conductive graphene inks have been widely adopted for their versatility in printing (e.g., inkjet, screen, gravure, and offset) and lithography techniques. Printing graphene enables cost-effective and high-throughput flexible electronics production, but often requires excessive post-processing, such as temperature treatment. For relatively high temperature resistant flexible substrates (e.g., poly­(ethylene naphthalate) (PEN, 262–275 °C melting temperature), polyimide (PI, decomposes >260–315 °C) or polyethylene terephthalate (PET, 245–260 °C melting temperature)), the temperature treatment range can change from 150 to 200 °C depending on the melting temperature. The temperature range could be elevated more (300 to 950 °C) for high-temperature-resistant rigid substrates, such as glass, to enable annealing. Since the printing can be done directly on the flexible substrate in a relatively large area, graphene transfer is not often required. However, in case of need, the printed graphene patterns could be transferred to another flexible substrate using polymer or thermal tape-assisted transfer procedures.^4,7,15^ Other graphene transfer methods include using adhesive tapes, transfer printing, and micro transfer molding.^8,9,16^ All of these methods involve multiple steps and certain limitations. For instance, the adhesive tape method suffers from resolution and feature size limitations affecting the efficient transfer.^17^ Transfer printing requires preemptive mold etching, stamping, and surface modification of molds,^18^ while the micro transfer molding also requires preemptive molding as well as accurate filling, and vacuum drying.^19^

Overall, the currently available graphene-based flexible electronics fabrication methods require multiple steps involving harsh chemical, thermal, and physical treatments. Most of these processes are not feasible for Food and Drug Administration (FDA) approved biodegradable polymers (e.g., polylactic acid (PLA), poly­(L-lactic acid) (PLLA), polycaprolactone (PCL), or poly­(lactic-*co*-glycolic acid) (PLGA)) with low glass transition temperature (range – 65 to 65 °C) and low processing thermal stability.^8,9,16,20^ Therefore, the current state of the art in flexible electronics fabrication methods needs additional improvements to develop a graphene-based biodegradable and implantable flexible electronic device that can modulate the damaged cells/tissues and enable regeneration, repair, and functional recovery.

This study mainly focuses on developing an alternative fabrication method to achieve graphene-based biodegradable and implantable flexible electronic devices for cell/tissue modulation. The fabrication method effectively combines graphene ink preparation, polymer film casting, high-throughput laser cutting, and water immersion-based large-area graphene transfer (Scheme 1). The method starts with the preparation of conductive graphene ink with the desired rheological properties for casting (Scheme 1A). The ink is then used to create a multilayer conductive graphene sheet on a glass substrate (A4-size) using an automated film applicator with controlled wet casting thickness. Upon drying, the graphene sheet on the glass substrate is subjected to temperature treatment to evaporate solvents and improve structural and electrical properties. At this point, low-temperature treatment (100–150 °C, generally classified as baking <200 °C) is applied to facilitate the transfer process, though it is also possible to apply high-temperature annealing (300 to 950 °C) on the glass substrate. Once the temperature-treated graphene sheet is obtained, a polymer solution (e.g., FDA-approved biodegradable and implantable PLA) is cast on top of the graphene sheet using an automated film applicator with controlled wet casting thickness. After room-temperature drying and film formation, the polymer/graphene film is immersed in deionized (DI) water at room temperature and naturally lifted off from the glass substrate through work of adhesion and hydrophilic/hydrophobic interactions, enabling large-area graphene transfer (Scheme 1B). The large-area graphene transfer method is further integrated with high-precision (minimum feature size is 50 μm, movement and engraving resolutions are 10 μm) and high-throughput laser cutter to fabricate large-area graphene-based patterns. After temperature-treated graphene sheet formation, the pattern of interest is obtained via precise laser engraving. Then, the same polymer casting and water immersion-based lift-off method is applied to transfer large-area graphene patterns to the flexible polymer film surface (Scheme 1C).

**Scheme 1:**
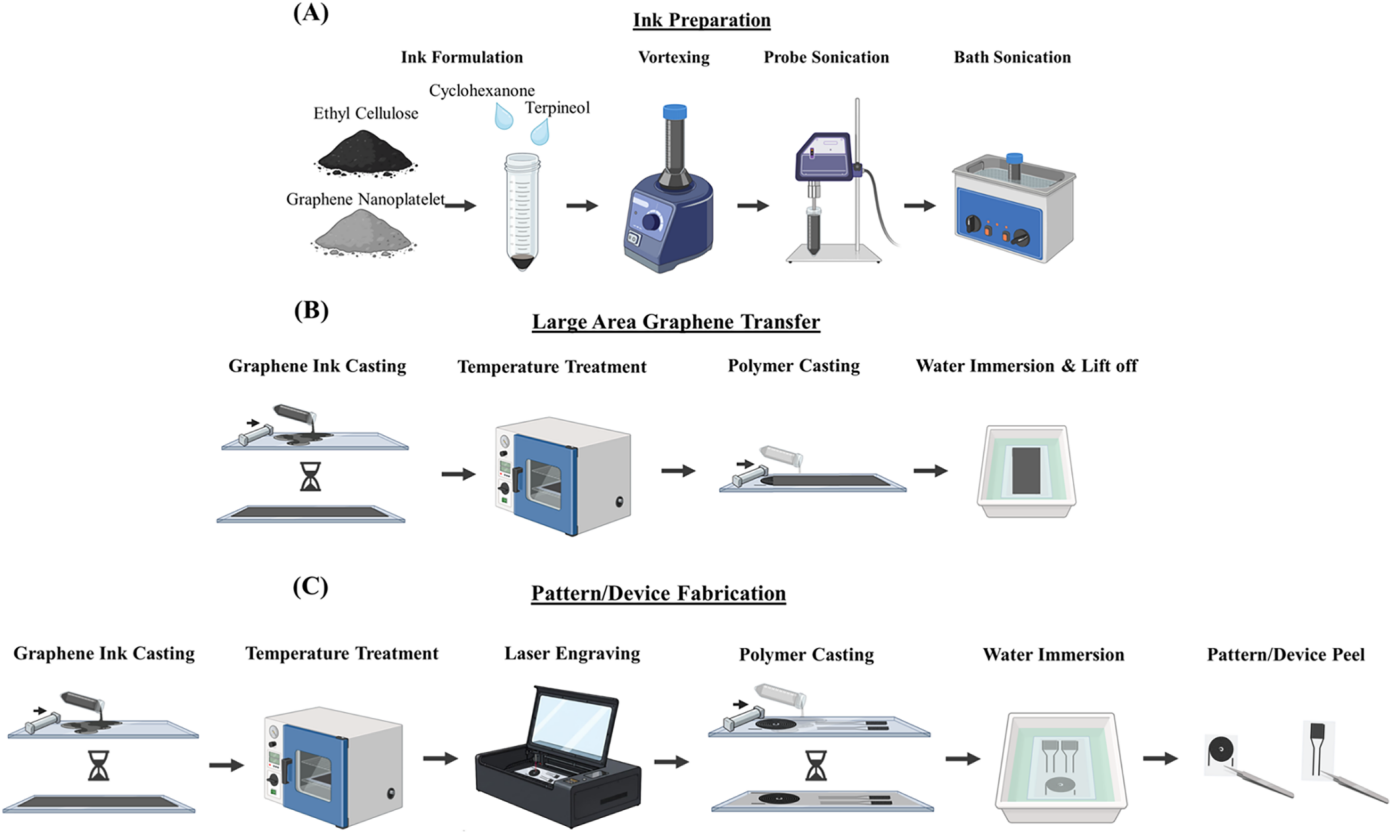
Illustration Diagram for Fabrication Process^a^

a (A) Ink preparation, (B) large-area graphene transfer process, and (C) pattern/device fabrication process. The scheme was generated using BioRender. For the casting bar and laser engraving machine, Google AI was used to convert real images into cartoon format. The scheme was then organized in PowerPoint.

With this method, we demonstrated successful large-area graphene sheet (Scheme 1B) or pattern (Scheme 1C) transfer from the glass substrate to the flexible and biodegradable polymer substrate. This method resulted in complete (∼100% yield) detachment of the graphene/polymer layered material from the glass substrate without leaving any fraction of the cast and temperature-treated graphene sheet (or pattern) behind after water immersion. The obtained graphene/polymer-based flexible electronic films were characterized in terms of structure, integrity, electrical properties, and stability. The initial material-cell interactions and in vitro cytocompatibility were also assessed.

This fabrication method is envisioned to be used to develop graphene- and polymer-based biodegradable and implantable flexible electronic devices to stimulate neural cells and tissues (Scheme 2). For this purpose, two functional devices were fabricated to demonstrate proof of concept: (1) a graphene coil-integrated electrode cuff (Scheme 2A) and (2) an interdigitated (IDE) graphene cuff-integrated piezoelectric device (Scheme 2B). The electrical functionality as well as the ex vivo handling and implantation/suturing feasibility of these devices were tested. The presented results indicated that the fabricated materials/devices have structural integrity and stability, possess desired electrical properties, show complete in vitro cytocompatibility, and are feasible for in vivo implantation.

**Scheme 2:**
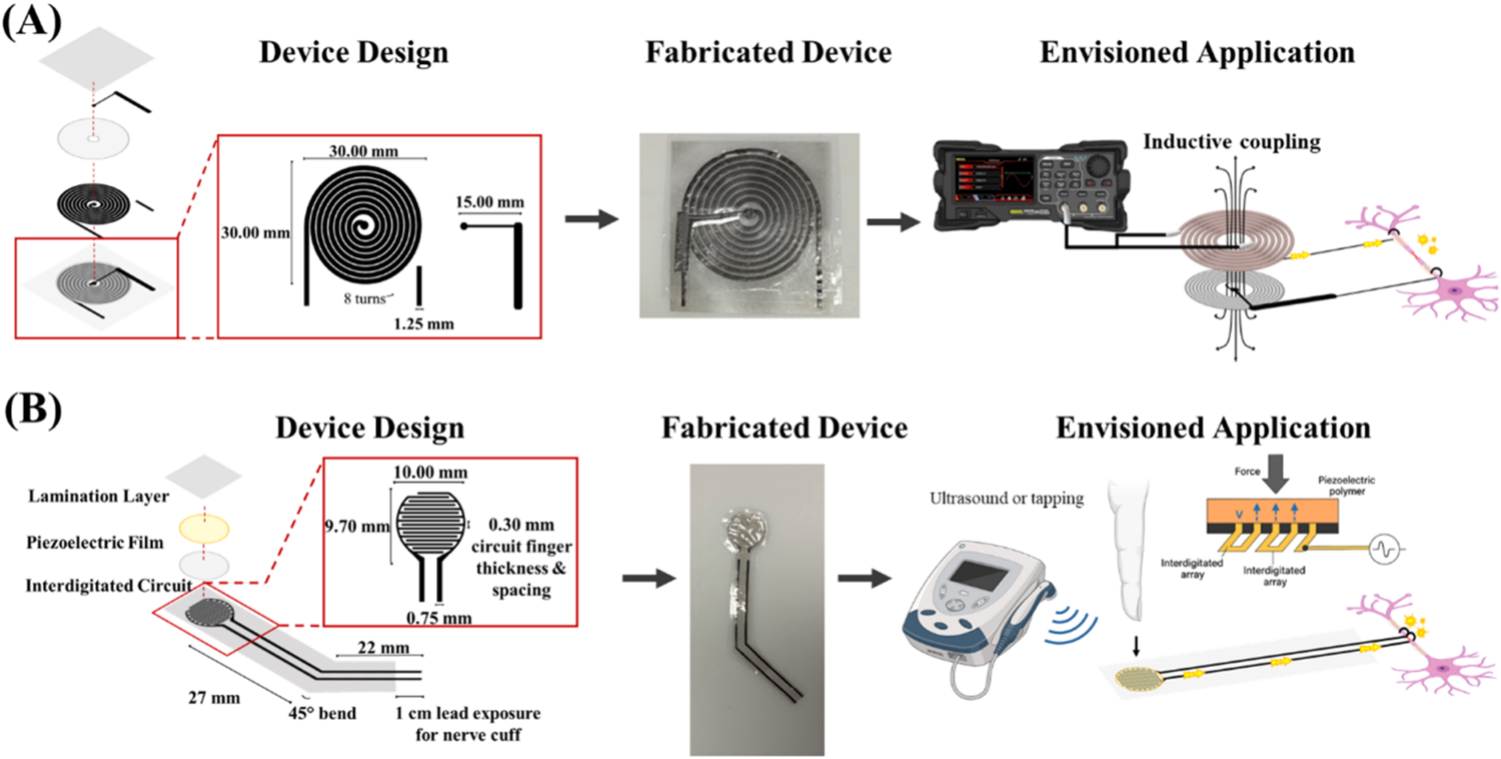
Illustration Diagram for the Fabrication of Functional Devices^a^

a (A) Graphene coil-integrated electrode cuff and (B) interdigitated graphene cuff-integrated piezoelectric device. The scheme was generated using BioRender. For the oscilloscope, Google AI was used to convert a real image into a cartoon format. The scheme was then organized in PowerPoint.

Overall, this study emphasizes the importance of this newly developed alternative fabrication method in obtaining biodegradable and implantable flexible electronic devices and lays the groundwork for future studies focusing on implementing fabricated devices for neural tissue regeneration applications. On a broader scale, the use of this newly developed room-temperature-operated, cost-effective, high-precision, high-throughput, water immersion-based large-area graphene transfer method could pave the way for developing functional devices targeting various applications, including healthcare monitoring, wearable electronics, sensors, and implantable devices.^21–23^

## 2 Experimental Section

### 2.1 Graphene Ink Preparation

To prepare 20 mL of graphene ink, graphene nanoplatelets (500 mg, ACS Material #GNNP0051—nanoscale particles of graphite with very small stack size (2–10 nm thickness and 2–7 μm diameter)) and ethyl cellulose (500 mg, viscosity 100 cP, 5% in toluene/ethanol 80:20, Sigma-Aldrich Catalog no. 247499 used as binder and stabilizing agent) were dissolved in 17 mL (85 v/v%) cyclohexanone (Sigma-Aldrich, Cat. no. 398241) and 3 mL (15 v/v%) terpineol (Sigma-Aldrich #86480). After the addition of each component (i.e., graphene nanoplatelets and ethyl cellulose), the graphene ink was vortexed and probe-sonicated (1 min at 100% amplitude × 3 times). Then, the ink solution was placed in a bath sonicator at 70 °C for 24 h to break down the graphene into smaller particles. During this period, intermittent vortex and probe sonication were performed to enhance the uniform dispersion. After the initial bath sonication step, the solution was vortexed for an additional 5 min and underwent another probe sonication (1 min at 100% amplitude × 3 times), followed by a second bath sonication cycle for 24 h at 70 °C (Scheme 1A). Finally, another round of vortex and probe sonication was performed, and the uniform ink solution was transferred to a new centrifuge tube.

The rheological characteristics of the ink were assessed using a modular compact rheometer (Anton Paar-MCR301). Shear stress versus shear rate and viscosity versus shear rate measurements were conducted using a CC27/AL measurement system with standard concentric cylinder (bob and cup) geometry. A steady-state flow sweep was performed to determine the viscosity as a function of the shear rate. The shear rate was logarithmically ramped from 0.01 to 100 s^–1^, and viscosity–shear rate curves were generated from the steady-state data using Ostwald–de Waele (power-law) model.

### 2.2 Graphene Transfer and Flexible Electronic Fabrication Process

After the ink solution was prepared, multiple layers of graphene (1 to 3 layers) were cast onto a glass substrate (A4-size) using an automated film applicator (TQC Sheen, Cat. #AB3652). Each layer had a 400 μm wet casting thickness and was cast after the previous layer was fully dried at room temperature. The purpose of applying multiple layers of graphene ink solution was to obtain a uniform and continuous graphene sheet with efficient conductivity. Once a completely dry uniform graphene sheet was obtained (generally after applying 3 layers of graphene ink solution), it was subjected to low-temperature treatment (at 100 and 150 °C) for 24 h to enhance electrical and surface properties.

To obtain a large-area conductive graphene sheet in the form of a flexible film, polylactic acid solution (11 wt % PLA (Ultimaker filament Cat. #1614) in chloroform (Fisher Scientific, Cat. #C6074)) was cast on top of the graphene sheet using a casting knife with 750 μm wet casting thickness and dried at room temperature overnight. Then, the obtained graphene/PLA film on the glass substrate was submerged in DI water to transfer the graphene sheet to the PLA substrate through lift-off (Scheme 1B). We specifically focused on using PLA because it was already approved by the FDA for biomedical applications; however, this method could also potentially be applied to other non-water-soluble biodegradable polymers (e.g., PLLA, PLGA, or PCL).

To obtain flexible electronic patterns with specific geometry, feature size, and dimension, the obtained graphene sheet on the glass substrate (3 layers of graphene ink solution casting followed by low-temperature treatment) was engraved into the desired pattern design using an ×Tools Laser Cutter (power: 40%, speed: 300 mm/s, movement and engraving resolutions: 10 μm, lines/cm: 220 corresponding to minimum feature size of ∼50 μm). The pattern design was created in Inkscape using an inverted. svg file to etch away the undesired graphene, leaving the desired pattern design to be revealed on the glass substrate. After the laser engraving and pattern preparation was complete, the PLA solution was cast, dried, and water-immersed for graphene sheet transfer through lift-off as described above (Scheme 1C).

### 2.3 Structural Characterization

The cross section and surface structure of the transferred graphene sheet before and after temperature treatment were assessed via scanning electron microscopy (SEM) (Thermo Fisher Apreo 2S). The samples were sputter-coated with platinum before the analysis, and the images were taken using secondary electron mode. The thickness of the graphene and PLA layers as well as the overall film thickness were evaluated using cross-sectional SEM images via image analysis software (ImageJ). The overall film and sheet layer thickness was also confirmed by using a stylus profilometer (Sloan Dektak 3030ST).

The surface roughness of the graphene (untreated and temperature-treated on the PLA substrate) was evaluated using a profilometer (Keyence VR-6200 3D Optical Profilometer), while the hydrophilicity/hydrophobicity of the graphene surface (untreated and temperature-treated on the PLA and glass substrate) was assessed by contact angle measurement. Briefly, a 10 μL droplet of DI water was deposited onto the samples at room temperature and contact angle measurements were recorded using a Ramè-Hart Contact Angle Goniometer.

X-ray photoelectron spectroscopy (XPS) (PHI VersaProbe 5000 X-ray Photoelectron Spectroscopy) was used to assess the effect of temperature annealing and stability of the graphene structure on the PLA substrate. A monochromatic Al Kα X-ray source (1486.6 eV) was used in XPS analysis with an electron takeoff angle of 45° from a normal sampling surface. Survey scans were collected from 10 to 1100 eV with a pass energy of 187.85 eV.

X-ray diffraction (XRD, Rigaku SmartLab) patterns of graphene on PLA substrate were recorded using a standard goniometer equipped with an XY-4 in. φ attachment head (scan axis θ/2θ). An X-ray generator of 40 kV and 45 mA was used with 1D scan mode. Data were collected over the 2θ range of 10–80° with a step size of 0.02° and a scan rate of 2.00 °/min.

Raman spectra of graphene on PLA substrate were collected using a Renishaw In Via Raman spectrometer using a 532 nm laser, 20× objective lens, and an average over 50 accumulations. Spectra were acquired in the range of 1000–3000 cm^–1^ with a spectral resolution of 1 cm^–1^. The characteristic D, G, and 2D bands were analyzed to assess the structural disorder, layer number, and overall graphitic quality.

### 2.4 Mechanical Properties

Tensile testing was conducted to evaluate the mechanical properties of graphene/PLA films using a universal testing machine (Instron Premium 5969 Mechanical Testing System). Specimens were prepared in rectangular geometry according to ASTM D882–12 guidelines (dimensions: 5 cm long and 1 cm wide) with a strain rate of 5 mm per minute. Mechanical parameters including ultimate tensile strength, Young’s modulus, and elongation at break were determined using the obtained data.

A 90° peel test was also performed to quantify the adhesive strength between the glass substrate and graphene/PLA film (temperature-treated graphene sheet interfacing with glass) for both dry and water-immersed samples. The sample dimensions were 4 cm in width and 20 cm in length. The glass substrate with cast graphene/PLA film on it was mounted at a fixed position, and the free end of the adhered graphene/PLA film was peeled vertically from the glass substrate at a constant crosshead speed of 10 mm/min, maintaining a 90° angle throughout the test. Force–displacement data were recorded continuously, and the peel strength was calculated as the average steady-state force normalized to the specimen width.

### 2.5 Sheet Resistance and Stability

The sheet resistance of the graphene on PLA substrate (1, 2, and 3 layers, untreated and temperature-treated) was determined using a four-point probe (Kaivo KV-FP-001, measures sheet resistance based on single point current (I) and voltage (V)) to observe the effect of the layers and temperature treatment on electrical conductivity. The *I*–*V* curve of the graphene on glass and PLA substrate was also measured by using a custom-designed probe station (Micromanipulator 450PM-B integrated with a Keithley 2636B SMU) by sweeping the current and measuring the voltage.

The bending and washing stability of the graphene/PLA films (3-layer graphene ink solution temperature-treated at 100 °C) were also determined. Bending tests were performed as periodic bending cycles over a fixed curvature, with sheet resistance measured after each cycle. Additionally, the stability of the graphene/PLA films was assessed by submerging them in Dulbecco’s phosphate buffered saline (DPBS) (Gibco Cat. no. 14190144) and measuring their sheet resistance before and after washing for 7 days. The in vitro stability of the graphene/PLA films was also assessed by immersing the films in DPBS for 90 and 22 days. The changes in weight, pH, and sheet resistance were measured before and after the immersion. The stability and degradation of the graphene/PLA films for potential in vivo applications were also assessed via accelerated degradation test in simulated body fluids (0.9 wt % NaCl at 72 °C for 72 h as stated in ASTM (ISO 10993–13)) and enzyme degradation test (using myeloperoxidase (MPO, 100 μM) enzyme in the presence of hydrogen peroxide (H_2_O_2_, 200 μM) for 72 h at 37 °C). The sheet resistance and sample weight were measured before and after the tests. The structural changes after the incubation period were also evaluated by SEM imaging and XPS analysis.

### 2.6 Material-Cell Interactions

The material-cell interaction and in vitro cytocompatibility of the patterned graphene/PLA films were tested on primary human Schwann Cells (hSCs) and human adipose-derived mesenchymal stem cells (hAD-MSCs). These cells were specifically selected: (1) because of our interest in peripheral nerve injuries and the important role of these cells in peripheral nerve regeneration and (2) to demonstrate that the developed materials can be used as an interface enabling a favorable environment for cells to attach, grow, and proliferate.

#### 2.6.1 Primary Human Schwann Cells (hSCs) Culture and Maintenance

Primary human Schwann cells (hSCs) were obtained from iXCells Biotechnologies and maintained in a basal cell culture medium with an enhanced growth kit (iXCells Biotechnologies, San Diego, CA; Cat. #MD-0055). Prior to hSCs seeding, the culture plates were precoated with 0.01% poly-l-lysine solution (Sigma-Aldrich, St. Louis, MO; cat. no. P4707) by following a modified version of the supplier’s coating protocol. Briefly, culture plates were treated with the recommended coating volume (1 mL per 25 cm^2^) for 1 h at 37 °C. After incubation, the plates were left to dry in the biosafety cabinet at least 2 h before cell seeding. The hSCs were passaged every 5–7 days using 0.25% Trypsin-EDTA (Gibco, Waltham, MA; Cat. #25200056). Typically, cells were seeded at a splitting ratio of 1:2 or 1:3 upon reaching 70% confluency, and medium was changed every 2 days.

#### 2.6.2 Human Adipose-Derived Mesenchymal Stem Cell (hAD-MSCs) Culture and Maintenance

Human adipose-derived mesenchymal stem cells (hAD-MSCs) obtained from ATCC (PCS-500–011) were cultured in mesenchymal stem cell basal medium (ATCC, PCS-500–030) containing 2.1% of MSC supplement (ATCC, PCS-999–038), 1.2% of l-alanyl-l-glutamine (ATCC, PCS-999–034), and 0.1% of penicillin-streptomycin amphotericin B solution (ATCC, PCS-999–002) in a sterile 75 cm^2^ cell culture flask, kept in an incubator at 5% CO_2_ and a temperature of 37 °C. Typically, cells were seeded at a splitting ratio of 1:2 or 1:3 upon reaching 70% confluency, and medium was changed every 2 days.

#### 2.6.3 Cell Viability

To assess the initial effect of patterned graphene/PLA films on cell viability, a Live/Dead cell assay (Live/Dead Viability/Cytotoxicity Cell Imaging Kit, Life Technologies, Cat. #R37601) was performed by following the manufacturer’s instructions. Briefly, the patterned sample was sterilized by applying ultraviolet (UV-light) and fixed onto autoclaved 9 mm microscope coverslips using a sterile surgical glue. Then, the cells were harvested and seeded on the patterned sample (5 × 10^4^ cells per sample, poly-l-lysine coating was applied to the sample material for hSCs while no coating was applied for hAD-MSCs). The Live/Dead cell assay was performed, and fluorescence microscopy (Nikon Eclipse Ts2R-FL) images were taken after 7 days of incubation at 37 °C with 5% CO_2_.

For more quantitative analysis, a cell proliferation assay (CellTiter 96 AQueous One Solution Cell Proliferation Assay, Promega, cat. #G3580) was also performed by following the manufacturer’s instructions. Briefly, the samples were prepared and sterilized, and cells were seeded (1 × 10^4^ per sample) as described above. After 3 days of incubation, the assay was performed and the absorbance of the collected cell culture media was measured at 490 nm using a microplate reader (BioTek Synergy HTX). In all experiments, cells cultured in tissue culture plates without sample material were used as a positive control.

#### 2.6.4 Cell Cycle and Apoptosis

Cell cycle and apoptosis analysis were performed as described before.^24^ Briefly, the samples were sterilized, and the cells were seeded as described above (2 × 10^5^ per sample). For cell cycle analysis, after 7 days of incubation, cells were harvested and washed with cold phosphate buffered saline (PBS) via centrifugation at 200*g* for 5 min. Then, the cells were fixed with 70% v/v ethanol under continuous vortexing and washed 3 times with PBS via centrifugation. The cell pellet was resuspended in 1 mL of PBS containing 0.1% Triton X-100. Subsequently, 200 μg/mL RNase was added, and the cells were incubated at 37 °C for 30 min. After incubation, 100 μg/mL propidium iodide (PI) (Molecular Probes, Cat. #P3566) was added to stain DNA, and the mixture was incubated in the dark at room temperature for 15 min and analyzed using BD FACS Canto II flow cytometry. For cell cycle analysis, data were analyzed by using FlowJo software to determine the distribution of cells across the G0/G1, S, and G2/M phases.

For apoptosis analysis, the Annexin V conjugates for apoptosis detection (Invitrogen, cat. no. A13201) were used according to the manufacturer’s instructions. In brief, after harvesting and washing, cells were resuspended in 1X annexin-binding buffer at a density of approximately 1 × 10^6^ cells/mL. A total of 100 μL of the cell suspension was incubated with 5 μL of FITC Annexin V and 1 μL of a 1000 μg/mL working solution of PI. The cell suspension was incubated in the dark at room temperature for 15 min. After incubation, 400 μL of 1X annexin-binding buffer was added, and samples were analyzed using a BD FACS Canto II flow cytometer. For apoptosis, fluorescence emission at 530 nm (FL1) for FITC Annexin V and > 575 nm (FL3) for PI was measured. Cell populations were categorized as live, apoptotic, or dead based on fluorescence intensity.

#### 2.6.5 DNA Damage

To further evaluate the impact of the patterned graphene/PLA films on DNA stability, comet assay (Abcam, Cat. #238544) was performed following the manufacturer’s instructions. The samples were sterilized; the cells were seeded as described above (2 × 10^5^ per sample) and incubated for 7 days. All necessary solutions were prepared prior to the assay, as instructed. Cells were harvested, centrifuged, and resuspended at a concentration of 1 × 10^5^ cells/mL in ice-cold PBS. Then, samples were lysed as described in the protocol. Following sample lysis, electrophoresis was carried out using TBE buffer at 13 V for 15 min. Afterward, the images of samples were taken by a fluorescence microscope (Nikon Eclipse Ts2R-FL) to assess DNA damage.

#### 2.6.6 Immunocytochemistry

Immunofluorescence staining was performed following a previously established protocol.^24^ The samples were sterilized, and the cells were seeded as described above (2 × 10^5^ per sample). After 7 days of incubation, cells were fixed in cold 4% paraformaldehyde fix solution (PFA) for 20 min, followed by three gentle washes in PBS. Then, cells were permeabilized in blocking buffer containing PBS with 0.2 v/v% Triton X-100, 10 v/v% donkey/goat serum, and 0.4 wt % BSA at room temperature for 1 h, followed by three PBS washes. After washing steps, samples were incubated overnight at 4 °C with primary antibodies (calcium binding mouse-α-S100β (Abcam-ab11178), rabbit myelin basic protein (MBP) (Invitrogen MA5–15922), and stem cell markers mouse CD29 (Integrin β 1) (Invitrogen 14–0299–82) and mouse CD44 (type 1 transmembrane glycoprotein) (Invitrogen 156–3C11)) diluted 1:200 in blocking buffer. The following day, samples were washed with PBS and then incubated at room temperature with secondary antibodies (goat-α-mouse and rabbit AlexaFluor488 Cy3 (1:500, Invitrogen A32723 and A32731)) and DAPI (4′,6-diamidino-2-phenylindole, Invitrogen) diluted 1:400 in blocking buffer for nucleic acid staining. Finally, stained cells were visualized using a fluorescence microscope (Nikon Eclipse Ts2R-FL- Excitation/Emission Max 493/518 nm).

### 2.7 Functional Device Fabrication and Testing

Two functional devices; (1) a graphene coil (8 turns, 30 mm diameter, 1.25 mm feature width, 300 μm spacing) integrated electrode cuff (Scheme 2A), and (2) an interdigitated graphene cuff-integrated piezoelectric device (16 fingers, 10 mm width, 750 μm feature width, 300 μm spacing and 300 μm finger width) (Scheme 2B), were fabricated using the developed method. The inductive coupling frequencies between the graphene coil (secondary) and custom-designed primary coil were found by sweeping the frequency systematically and identifying significant decibel (dB) peaks using a network analyzer. The graphene coil was placed within the center of the primary coil, which was connected to a function generator, and the frequency and voltage output were recorded using an oscilloscope.

To assemble the interdigitated (IDE) graphene cuff-integrated piezoelectric device, the graphene-based IDE was fabricated, as mentioned above. The chitosan-based piezoelectric component of the system was fabricated by following previously described methods.^25^ The IDE and piezoelectric component were cut to the same size and combined by heat pressing (at ∼65.5 °C for several minutes) to form the complete device. Then, the exposed leads of the device were attached to an oscilloscope to read the frequency and output voltage as a result of a finger tapping test, in which finger pressure was regularly applied on and off at the surface of the piezoelectric portion of the platform.

### 2.8 Device Implantation Feasibility

The implantation feasibility of the IDE graphene cuff-integrated piezoelectric device was evaluated on the pudendal nerve of a cadaver Sprague–Dawley rat, with implications for use in a stress urinary incontinence model, simulated in female rats using both bilateral pudendal nerve crush and vaginal distension injuries.^26,27^ The suturing and handling feasibility of the device was evaluated using previously established surgery techniques on the developed animal model. Only cadaver animals were used for these experiments to test the feasibility of suturing and implantation, and no other tests were performed on live animals.

### 2.9 Statistical Analysis

Statistical significance was evaluated using one- and two-way ANOVA analysis by Tukey’s method with a 95% confidence interval. At least three independent experiments were conducted for each analysis. Mean ± standard deviation is used to present the results.

## 3 Results and Discussion

The use of the graphene ink casting method to manufacture a multilayered conductive graphene sheet was successfully demonstrated in Figure 1A. The rheology results indicated that the prepared graphene ink exhibited Newtonian behavior with shear thickening, where viscosity increases as the shear rate increases and eventually becomes independent of the shear rate (Supporting Information, Figure S1). This behavior was contrary to conventional graphene inks, mainly used for printing applications and typically showing non-Newtonian, shear-thinning (pseudoplastic) behavior, where the viscosity decreases as the shear rate increases. The average viscosity of our graphene ink was around ∼0.0635 Pa.s. This value was significantly higher than the inks for inkjet or aerosol printing applications (ranging between ∼2–15 mPa.s) and closer to screen printing (ranging between ∼0.05–5 Pa.s), which is similar to our casting process.^28–30^ In addition, it was previously reported that the Newtonian behavior of the ink is desirable for the purpose of graphene casting as they tend to achieve a highly uniform sheet over large areas.^31^ Therefore, the rheological properties of our graphene ink formulation were suitable for the developed fabrication method.

**Figure 1:**
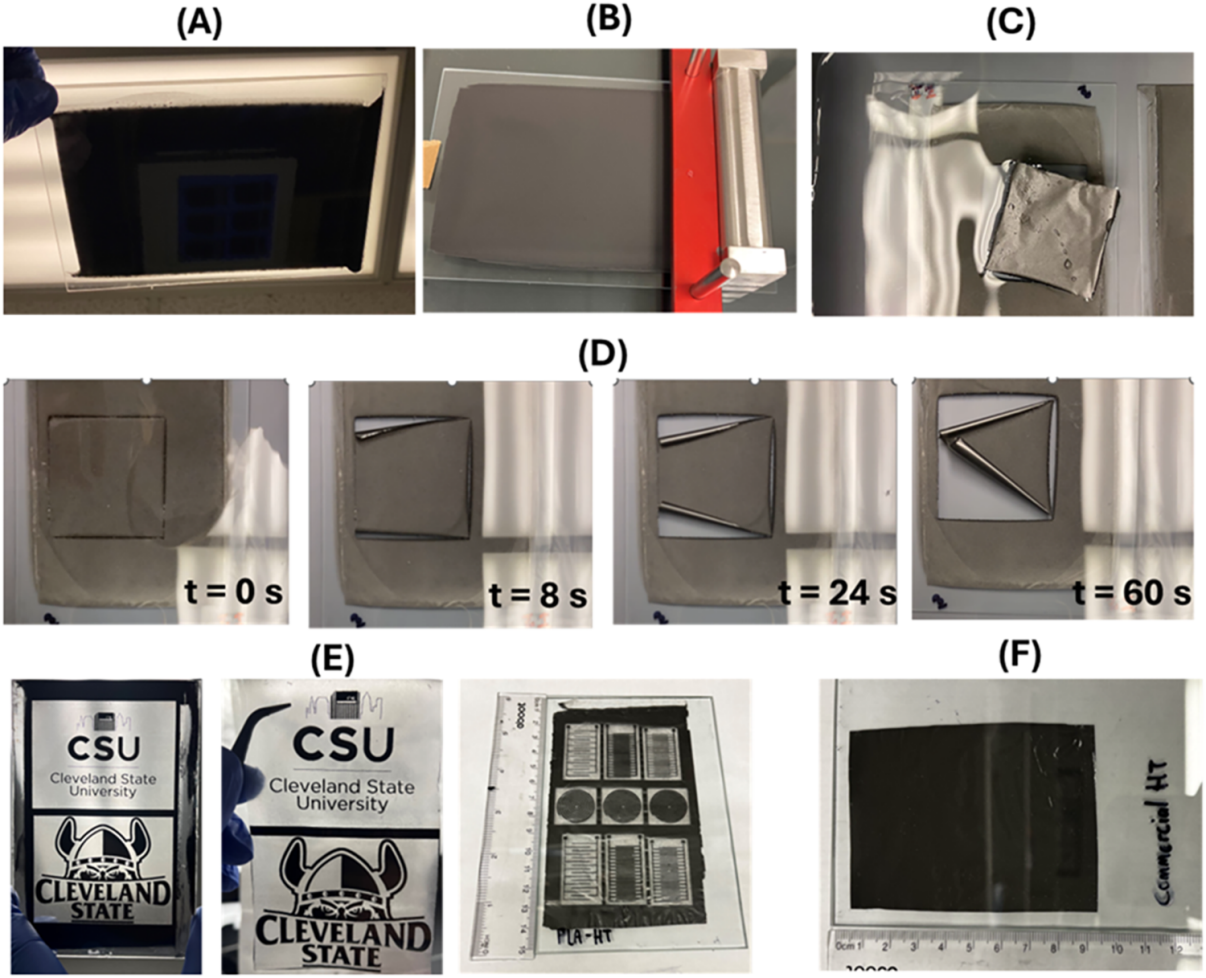
Large-area graphene transfer steps: (A) Casting of graphene ink on glass substrate. (B) Drying of graphene ink and casting another layer of PLA to form film. (C) Immersion in water to lift off the graphene/PLA-based conductive film. (D) Water immersion-based graphene transfer time lapse images. (E) Graphene/PLA-based flexible electronic CSU logo and patterns fabricated by combining laser engraving with the water immersion-based large-area graphene transfer method. (F) Water immersion transfer of commercial graphene ink-based conductive sheet.

The use of an automated film applicator enables precise control over the casting speed and wet casting thickness of the graphene ink. This enables casting of multiple layers and determines the homogeneous graphene ink distribution as well as uniform sheet formation on the glass substrate. In our previous studies, we used Teflon or Delrin molds/substrates to enable efficient graphene transfer by using their super-hydrophobic surface properties via polymer casting approach.^32,33^ Although the super-hydrophobicity of Teflon or Delrin substrate was helpful in terms of facilitating the graphene transfer, these substrates have preexisting surface patterns resulting from cutting and possess a nonflat surface, particularly for the large areas exposed to temperature treatment, which affect the uniformity of the obtained graphene sheet or pattern structure. Therefore, in this study, we used a glass substrate due to its smooth and pattern-free surface and resistance to high temperatures.

After drying and low-temperature treatment (mainly baking at low temperatures, < 200 °C), PLA solution was cast on top of the graphene sheet on the glass substrate (Figure 1B) to enable room-temperature-operated large-area graphene transfer without need for additional post-processing, as other existing methods require.^20,34^ Following the drying and PLA film formation at room temperature, the graphene/PLA film was immersed in DI water to facilitate large-area graphene transfer (Figure 1C). Here, we benefit from two phenomena: (1) the work of adhesion and (2) the hydrophobic behavior of graphene (enhanced after temperature treatment) to enable large-area graphene transfer from the glass substrate to the flexible PLA substrate. This method resulted in ∼100% yield in graphene transfer through detachment of the graphene/PLA layered material from the glass substrate without leaving any residue of the temperature-treated graphene sheet behind after water immersion.

In Figure 1C, the difference in the surface energies between graphene/PLA and graphene/glass interfaces upon the formation of films contributes to the complete transfer of graphene from the rigid glass substrate to the flexible PLA film. This can be theoretically calculated by using eq 1

1 $$W_{A-B}=4\left(\frac{\gamma_{A}^{d}\gamma_{B}^{d}}{\gamma_{B}^{d}+\gamma_{B}^{d}}+\frac{\gamma_{A}^{p}\gamma_{B}^{p}}{\gamma_{B}^{p}+\gamma_{B}^{p}}\right)$$

where γ^d^ and γ^p^ correspond to the dispersion and polar components of surface energy, where (γ = γ^d^ + γ^p^).^35–41^ For instance, the theoretically calculated work of adhesion between each interface supports the proposed hypothesis; *W*_Graphene‑Glass_ (65 mJ/m^2^) < *W*_Graphene‑PLA_ (87 mJ/m^2^).^37,39–44^ In addition, prescoring the area of interest to improve water penetration between the underlying graphene and glass interface further enhances the lift-off process due to the hydrophobic nature of graphene and lower work of adhesion at the graphene/water interface (*W*_Graphene‑Water_ (56 mJ/m^2^) < *W*_Graphene‑PLA_ (87 mJ/m^2^)).^37,39–43^

In addition to the above theoretical calculations, the adhesion energy of temperature-treated graphene sheet on glass substrate were also evaluated based on the results of the peel test (Supporting Information, Figure S2) for both dry and water-immersed samples using eq 2

2 $$\frac{P}{G_{A}}=\frac{1}{1-(\cos\alpha )}$$

where *P* (N/m) is peel force per width, *G*_A_ (J/m^2^) is the adhesion energy of the interface, and α is the peel angle. The adhesion of temperature-treated graphene sheet onto glass substrate after water immersion was calculated as 54 J/m^2^, while adhesion of same graphene sheet onto glass substrate in dry condition was calculated as 135 J/m^2^. Although the experimentally calculated adhesion energies are different from the theoretical ones, they align with the literature findings^45,46^ and demonstrate the significant difference between the dry and wet sample peeling. Hence, this experimental measurement also indicated that water immersion of the graphene samples facilitates lift-off for large-area graphene transfer. This also resulted in a time-efficient process (Figure 1D), especially by prescoring the area of interest to improve water penetration.

In this study, PLA was specifically selected because it is already approved by the FDA as an implantable and biodegradable material for biomedical applications. However, this process could also be applied to any other synthetic polymer that has the necessary surface energy and can be dissolved in inorganic solvents and not in water (i.e., cellulose acetate, PLGA, PLLA, PCL, or polystyrene). To demonstrate this capability, PLLA and polystyrene-based polymers were cast on the temperature-treated graphene sheet, and it was observed that both polymers formed a continuous film and were lifted off after water immersion, enabling 100% graphene transfer from the glass substrate to the film substrate without leaving any graphene residues on the glass (Supporting Information, Figure S3A,B).

This large-area graphene transfer method was further combined with precise and accurate laser engraving to obtain a graphene-based flexible electronic pattern/device via high-throughput fabrication. As seen in Figure 1E, any graphene pattern design with precise and accurate features can be created. Laser engraving provided the necessary power to directly remove graphene from the glass substrate, deriving the desired graphene pattern, which was transferred in the same manner as large-area graphene sheets with PLA film casting and water immersion. With this method, it is possible to obtain a minimum graphene feature size of ∼50 μm. In addition, this transfer method was not only restricted to our graphene ink formulation but also worked with commercially available conductive graphene inks (Graphene Works) as shown in Figure 1F.

The temperature treatment also influences the structure of the graphene, which, in turn, contributes to and facilitates the transfer process. As seen in Figure 2A–C, the cracks and discontinuities on the untreated graphene structure disappeared, forming homogeneous, smooth, and uniform graphene films as the treatment temperature increased to 100 and 150 °C. No significant differences were noted in terms of graphene structure for treatment temperatures of 100 and 150 °C. The PLA surface of the final film was dense and nonporous as seen in Figure 2D. Moreover, the defined graphene patterns and structure on the PLA substrate can also be seen in Figure 2E.

**Figure 2:**
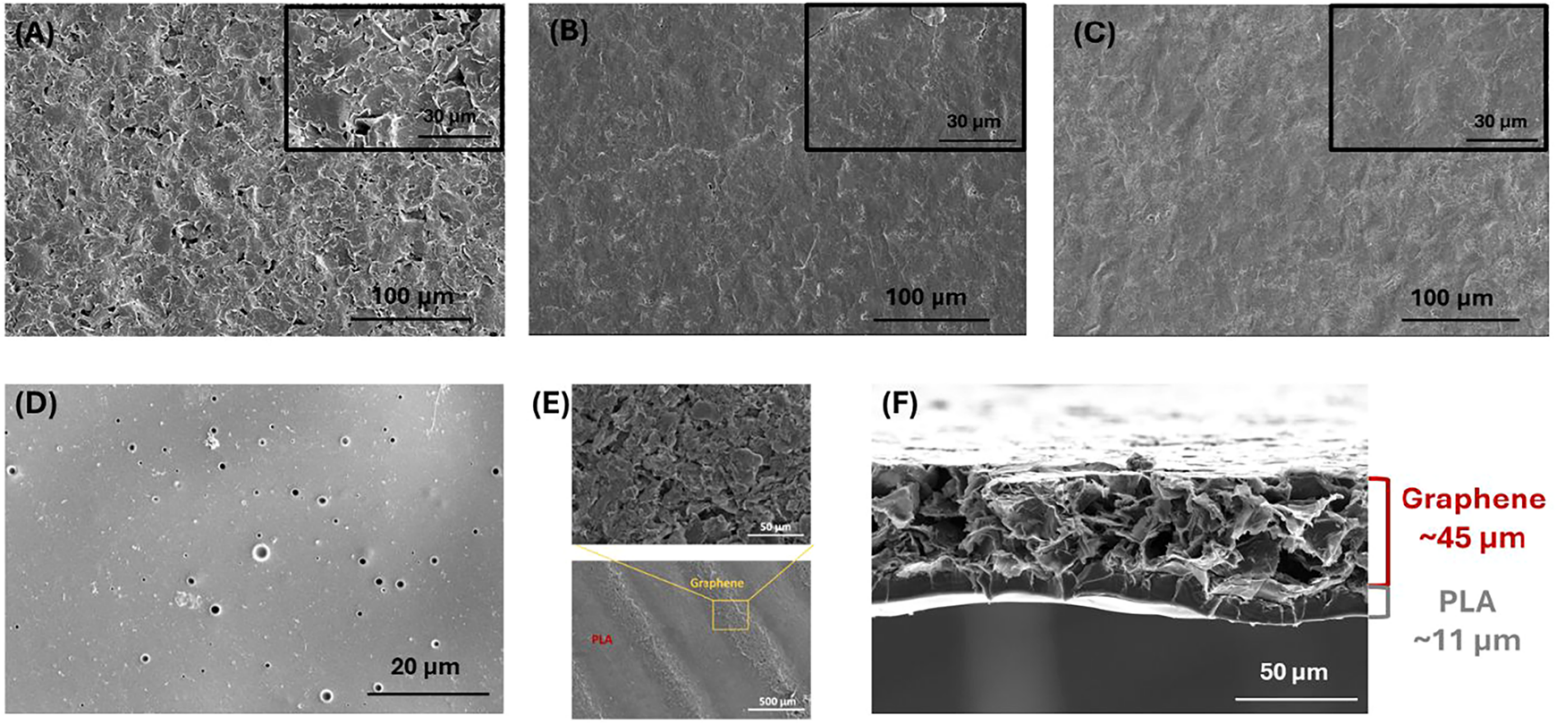
SEM images of (A) untreated graphene surface, (B) temperature-treated graphene surface at 100 °C, (C) temperature-treated graphene surface at 150 °C, (D) PLA surface, (E) graphene patterns on PLA substrate, and (F) cross section of graphene/PLA film.

The cross section image in Figure 2F confirms that PLA is completely integrated into the graphene, depicted as a stark contrast border between PLA and graphene layers. This also allows graphene side of the film to be highly conductive while the PLA side acts as an insulator. The overall dry thickness of the graphene/PLA film was around ∼65 μm based on the SEM cross-sectional images. The dry thickness of the graphene layer was ∼45 μm, while the PLA layer’s dry thickness was ∼11 μm. The overall dry thickness as well as the thickness of each layer (graphene and PLA) was also measured by a profilometer supporting the SEM results (Supporting Information, Figure S4A,B).

It was observed that temperature treatment reduced the roughness of graphene on PLA substrate, resulting in a relatively smoother surface (roughness decreasing from 10.58 to 3.99 nm) (Figure 3A–C, first row). In addition to the decrease in the roughness, the graphene surface on PLA substrate becomes slightly more hydrophobic with the effect of temperature treatment (contact angle increasing from ∼75̊ to ∼82̊) (Figure 3A–C, second row). The surface of the graphene sheet transferred onto the PLA substrate (Figure 2F) is the interface initially interacting with the glass substrate upon casting of graphene ink. Therefore, the surface properties at graphene/glass interface determine the effectiveness of the graphene transfer. To better understand this interaction, the contact angle of the graphene sheets on the glass was also evaluated (Supporting Information, Figure S5). It was observed that the temperature-treated graphene sheet on the glass substrate demonstrated higher hydrophobic behavior (contact angle ∼ 122̊) compared to the untreated graphene sheet on the glass substrate. It is noted that the graphene sheets on the glass substrate (both untreated and temperature-treated) demonstrated higher hydrophobicity (Supporting Information, Figure S5A–C) than the graphene sheets on the PLA substrate (Figure 3A–C, second row). This is mainly related to the hydrophilicity and hydrophobicity of the substrate material. After PLA casting on the graphene sheet, it is possible for PLA to diffuse into the graphene layer and reduce the hydrophobicity of the graphene surface. The contact angle of PLA film only was measured around ∼72̊ (Supporting Information, Figure S5D), which is close to the values presented in Figure 3A–C, second row. Therefore, the synergistic effect of reduced roughness and increased hydrophobicity of the graphene surface on the glass substrate upon temperature treatment contributed to the facile transfer of graphene via water immersion.

**Figure 3:**
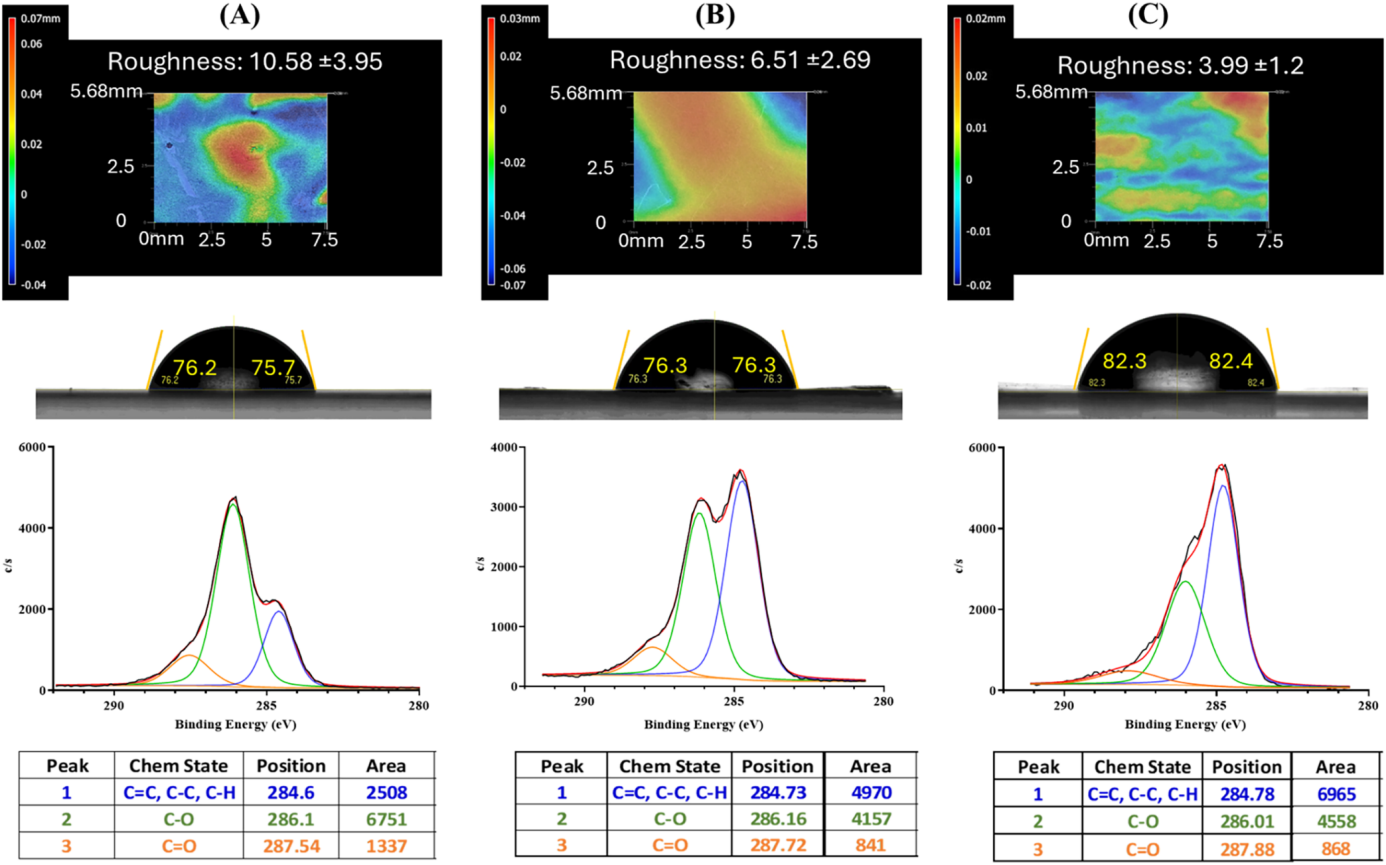
Surface topography/roughness (first row), contact angle (second row), and XPS (third/fourth row) of graphene surface: (A) untreated, (B) temperature-treated at 100 °C, and (C) temperature-treated at 150 °C.

The changes in roughness and hydrophobicity are also associated with alterations in the chemical structure of graphene upon the temperature treatment, as shown in the XPS results in Figure 3A–C, third/fourth rows. Temperature treatment was primarily used to evaporate the solvents in the graphene ink formulation to enable sheet formation and reduce the oxygen content in C–O and C=O bonds, leading to higher hydrophobic behavior.^47–49^ With temperature treatment, there was improvement in the reduction of both C–O and C=O bonds with the area of binding energy dropping by 35.07% in C–O and by 32.48% in C=O bonds for 150 °C and by 38.42% in C–O and 37.1% in C=O for 100 °C treatment. By freeing space held by oxygen, each annealed sample also improved C=C, C–C, and C–H bond area from 2,508 to 4,970 for 100 °C treatment and 6,965 for 150 °C treatment (Figure 3A–C, third/fourth rows). It was also noted that the samples keep their graphitic structure, showing a C 1s peak centered at 284.5 eV.

To support the XPS results, the XRD patterns and Raman spectra of graphene sheets treated at 100 °C were also analyzed. Temperature treatment at 100 °C was selected for XRD and Raman analysis (Figure 4) as there was no significant difference in the reduction of both C–O and C=O bonds between 100 and 150 °C treatment. The XRD pattern of the temperature-treated graphene sheet on PLA substrate demonstrated a graphitic structure (Figure 4A). This is characterized by a sharp, intense diffraction peak at 2θ ∼ 26.4̊–26.7̊, indicating a highly ordered crystalline structure with a small interlayer distance (*d*-spacing) (Figure 4A). Similarly, the Raman spectrum showed characteristic graphitic peaks (G peak ∼ 1600 cm^–1^, D peak ∼ 1350 cm^–1^, and a minor 2D peak ∼ 2700 cm^–1^, with the intensity of the G peak higher than the D peak) (Figure 4B). These observations indicated that the graphitic nature of the samples was preserved, albeit with the introduction of significant defects from the exfoliation or interlayer cleavage process. Graphene quality and structural defects were also determined by the Raman spectrum intensity ratio of the D band to the G band (*I*_D_/*I*_G_ = ∼0.25 for the untreated graphene and *I*_D_/*I*_G_ = ∼0.1 for the temperature-treated graphene). It was indicated that the temperature treatment resulted in achieving high quality and relatively low defect graphene (*I*_D_/*I*_G_ < 0.15) compared to the untreated graphene samples (*I*_D_/*I*_G_ > 0.15). It was also noted that observing the graphitic structure was because of the source of the graphene nanoplatelets used in these experiments. Graphene nanoplatelets obtained from ACS Material are nanoscale particles of graphite with very small stack size (2–10 nm thickness and 2–7 μm diameter). In addition, when compared with the untreated graphene sheets on PLA substrate, there is a significant increase in the intensity of the graphite peak in both XRD pattern and Raman spectrum, indicating the presence of larger graphitic domains with possibly improved stacking order of layers and crystal structure (Figure 4C,D).

**Figure 4:**
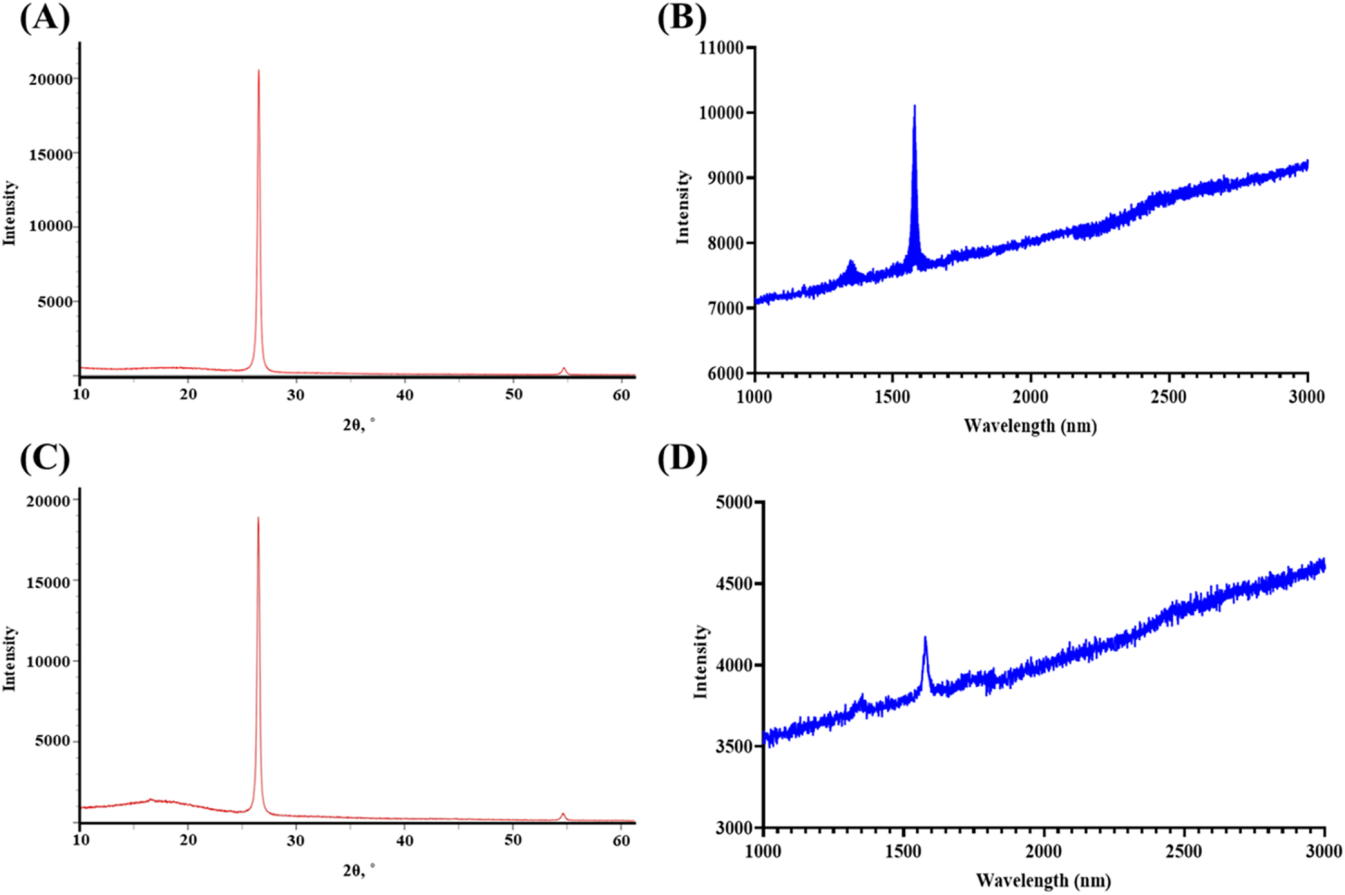
(A) XRD pattern and (B) Raman spectrum of graphene sheet temperature-treated at 100 °C on a PLA substrate. (C) XRD pattern and (D) Raman spectrum of untreated graphene on a PLA substrate.

The applied temperature treatment is relatively low (100 and 150 °C for 24 h) compared to other studies in the literature using harsher temperature annealing conditions.^47–49^ In addition, there was no significant difference between 100 and 150 °C treatment, in terms of surface roughness and hydrophobicity on the PLA substrate. For this reason, changes in the surface roughness and hydrophobicity upon the low-temperature treatment are relatively small compared to the untreated graphene control, particularly on the PLA substrate. Nevertheless, low-temperature treatment provided an easy 100% lift-off for the graphene after water immersion, while the untreated graphene required peeling off from the surface by applying force (Supporting Information, Figure S6A,B). For instance, when PLLA was used as a polymer layer, some parts of the graphene sheets were stuck on the glass substrate during the peel-off process, contrary to temperature-treated graphene, which provided 100% transfer via lift-off on PLLA (Supporting Information, Figure S3A). Similarly, easy lift-off of temperature-treated graphene on PLA substrate was shown in Figure 1C, while manual peel-off is required for untreated graphene sheet even after water immersion (Supporting Information, Figure S6B).

This is again due to the different surface energies of the graphene upon temperature treatment.^37,50^ The work of adhesion in the untreated graphene/water interface is relatively higher (*W*_Untreated Graphene‑Water_ (76 mJ/m^2^)) compared to the work of adhesion in the temperature-treated graphene/water interface (*W*_Treated Graphene‑Water_ (56 mJ/m^2^)) and closer to the work of adhesion in the graphene/PLA interface (*W*_Graphene‑PLA_, 87 mJ/m^2^). This is the main reason we observed easy lift-off with temperature treatment, while we needed to peel off the film from the glass substrate after water immersion for the case of untreated graphene (Supporting Information, Figure S6A,B).

It was also observed that the temperature treatment had a significant impact on the conductivity (or sheet resistance) of the graphene patterns (Figure 5A). This was mainly due to the reduction in C–O and C=O bonds and increase in C=C, C–C, and C–H bonds, as shown in XPS results (Figure 3A–C, third/fourth rows). In addition, the reduction in cracks and discontinuities on the untreated graphene structure and forming homogeneous, smooth, and uniform graphene sheets upon temperature treatment (Figure 2A–C) also resulted in an increased conductivity (or reduction in sheet resistance). In addition to the effect of temperature, increasing the number of layers also resulted in a decrease in sheet resistance due to the higher concentrations of graphene homogeneously dispersed by repetitive casts (Figure 5A). The combined effects of 3 layers of graphene and temperature treatment at 100 and 150 °C significantly decreased the sheet resistance to ∼40 Ω/sq (Figure 5A). The higher conductivity (or lower sheet resistance) could also be attributed to the graphitic nature of the samples, where the flexible graphite sheets generally exhibit sheet resistance values in the range of 30–3000 Ω/sq.^51–53^

**Figure 5:**
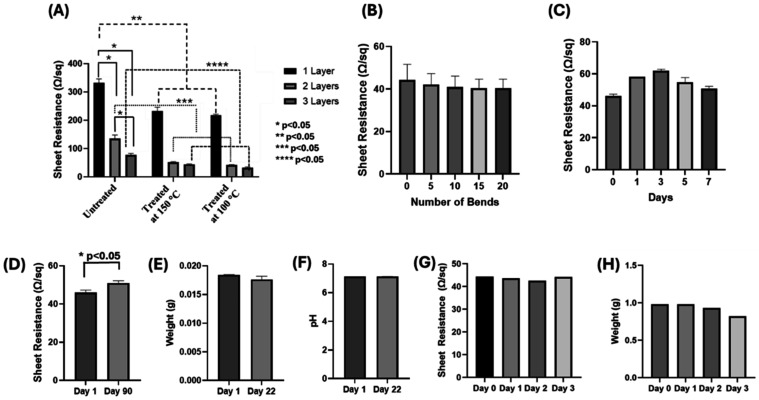
(A) Sheet resistance values of patterns with different numbers of graphene layers, treated for 24 h at 100 and 150 °C. (B) Washing and (C) bending stability of the graphene patterns prepared by 3 layers of graphene and treated at 100 °C. The effect of long-term in vitro degradation of graphene samples in PBS at pH 7.4 and 37 °C on (D) sheet resistance, (E) weight loss, and (F) pH of the environment. The change in (G) sheet resistance and (H) weight during the accelerated degradation test in simulated body fluid at 72 °C for 72 h showing the stability of the developed circuits. *, **, ***, and **** represent statistical significance (*p* < 0.05). The error bars are not visible in (F–H) due to the very minimal variability in the samples.

In general, sheet resistance of ∼40 Ω/sq was found to be homogeneous throughout the large-area graphene sheet transferred via water immersion. We also noted that there was no significant difference between the cases of 100 and 150 °C treatment conditions in terms of the obtained sheet resistance values, regardless of the number of layers (Figure 5A). Therefore, to reduce wasted energy, it was decided to maintain further temperature treatment at 100 °C for 24 h rather than 150 °C since similar sheet resistance values were obtained.

The I–V curve of the graphene sheets on glass and PLA substrate was also evaluated (Supporting Information, Figure S7). The graphene sheets (regardless of the temperature treatment and substrate material) displayed linear (Ohmic) behavior, indicating high electrical conductivity and low constant resistance. A similar trend in the change of sheet resistance upon temperature treatment was also observed in the *I*–*V* curves, where the resistance of the temperature-treated samples was lower than the untreated graphene sheets regardless of the substrate material (∼474 Ω/sq for untreated graphene sheet on glass and PLA substrate increasing to around ∼80 Ω/sq for the temperature-treated graphene sheets on glass and PLA substrate).

The patterned graphene/PLA films proved their integrity and stability by demonstrating maintenance of resistance through subjection of lengthy immersion in PBS and cyclic stress testing, with no significant difference in sheet resistance after these tests (Figure 5B,C). In addition, considering that the patterned graphene/PLA films will be designed and used as biodegradable and implantable devices to stimulate cells/tissues, their long-term in vitro integrity, stability, and degradation were also tested by incubating them in PBS solution at pH 7.4 at 37 °C for 3 months. The results indicated that there was no significant change in sheet resistance, weight loss, or pH of the environment (Figure 5D–F). In addition, no significant structural damage was observed after bending, washing, and incubation tests. The prepared graphene-PLA platforms also showed mechanical stability after long-term storage (Supporting Information, Figure S8A,B). The fresh sample had relatively smaller yield strength and elongation at break than the stored sample, while both have similar Young’s modulus (Supporting Information, Figure S8A,B).

In addition to the in vitro degradation, an accelerated degradation test was also performed in simulated body fluids at high temperatures to assess the stability of the fabricated patterns. The results indicated that at the end of 72 h incubation in simulated body fluid at 72 °C, no significant change was observed in the sheet resistance as well as the weight of the samples (Figure 5G,H). Similarly, when the circuits were incubated in myeloperoxidase (MPO) enzyme in the presence of H_2_O_2_ for 72 h at 37 °C, we also did not observe any significant change in the sheet resistance and the weight of the samples (data was not shown as it was the same as in Figure 5G,H). In addition, the structural changes in the samples before and after accelerated degradation and enzyme degradation were also evaluated using XPS and SEM imaging. No significant change was observed in the XPS analysis and SEM images after accelerated degradation (Supporting Information, Figure S9A,B) compared to the initial conditions (Figures 2B and 3B). On the other hand, increased oxidation on the graphene sample surface as well as structural changes were observed after the enzyme degradation tests (Supporting Information, Figure S9A,B). The surface oxidation and structural changes did not significantly affect the sheet resistance of the graphene samples, indicating stability; however, it also showed that enzyme-mediated oxidation-based biological degradation of the graphene/PLA structure is possible in the long run in vivo as mentioned in previous studies in the literature.^54–56^

Overall, possible patterns/devices fabricated using the developed method can be considered stable under harsh conditions and show potential for biodegradation over time. This is particularly valuable for implantable and biodegradable device development since such devices are expected to be stable during the treatment period and desired to be eventually degraded over time as the tissue is regenerated or an injury is healed.

PLA is FDA-approved and nontoxic for cells and tissues; however, there are conflicting reports about the biodegradability and biocompatibility of graphene-based materials. It has been recently shown that single-layer or multilayer graphene can be degraded by the body via specific enzymes (e.g., MPO) through enzymatic degradation,^54–56^ but toxicity is still a complex issue depending on various factors, such as size, shape, structure, fabrication method, and type of cells/tissues.^57–59^ Therefore, to make sure that the graphene-based flexible electronic patterns/devices fabricated via the developed method are not toxic, a detailed in vitro cytocompatibility analysis was conducted on two different cell types, primary hSCs and hAD-MSCs.

The results in Figure 6 indicated that the patterned graphene/PLA films did not alter the viability of hSCs (Figure 6A,B,E) and did not cause DNA damage or apoptotic cell population (Figure 6C,D,F). We also did not observe any alterations in cell cycle phases (Figure 6G,H). The cells on the patterned graphene/PLA films continue to express the primary Schwann cell markers, MBP and S100β, as seen in Figure 6I, similar to hSCs grown on regular petri dishes as controls.

**Figure 6:**
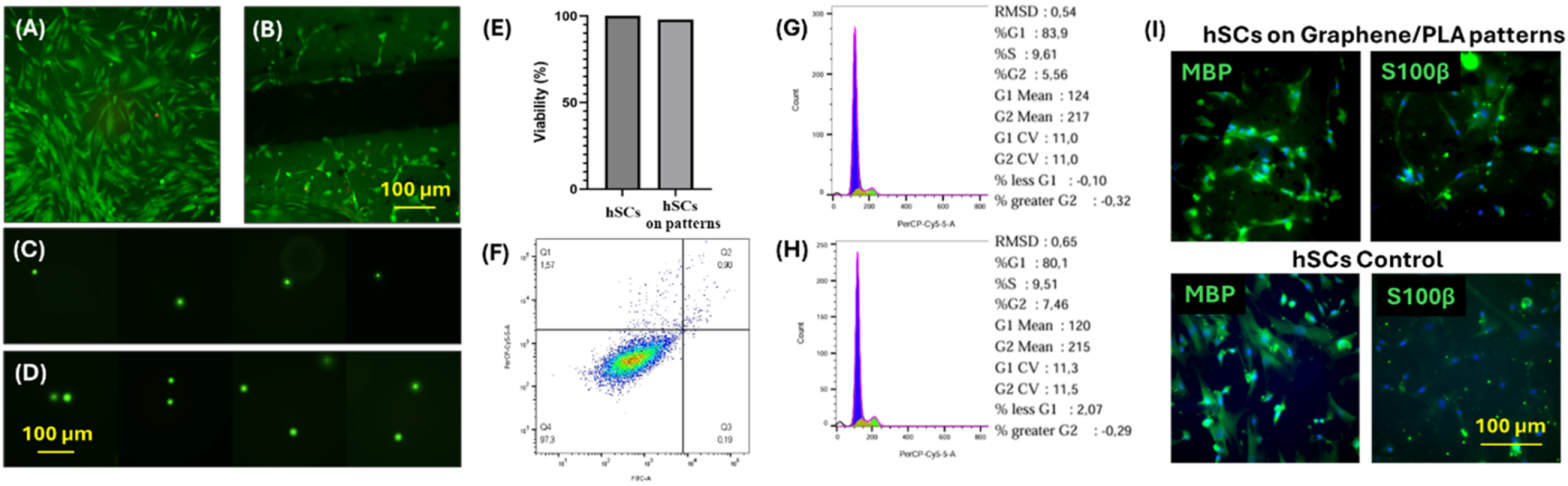
Live/Dead cell assay analysis at 7 days on (A) Control hSCs and (B) hSCs on patterned graphene/PLA films. DNA damage analysis using comet assay at 7 days on (C) Control hSCs and (D) hSCs on patterned graphene/PLA films. (E) Cell viability analysis of hSCs on patterned graphene/PLA films and control hSCs on Petri dishes at 7 days. (F) Apoptosis analysis on hSCs on patterned graphene/PLA films at 7 days. Cell cycle analysis on (G) Control hSCs and (H) hSCs on patterned graphene/PLA films at 7 days. (I) ICC analysis of hSCs markers, MBP, and S100β for hSCs growing on patterned graphene/PLA films, and Petri dishes (control) at 7 days.

The effect of the patterned graphene/PLA films on hAD-MSCs was also tested, and it was found that the pattern material did not have any adverse effect on hAD-MSCs viability, DNA damage, apoptosis, and cell cycle (Figure 7A–H). In addition, the cells continue to express their stem cell markers similar to those in the control case (Figure 7I). Therefore, our results indicated that the graphene-based flexible electronic patterns that were prepared with the developed fabrication method had no significant adverse effect on cells. These findings also show the potential in vivo biocompatibility of the future patterns/devices that will be developed by this fabrication method.

**Figure 7:**
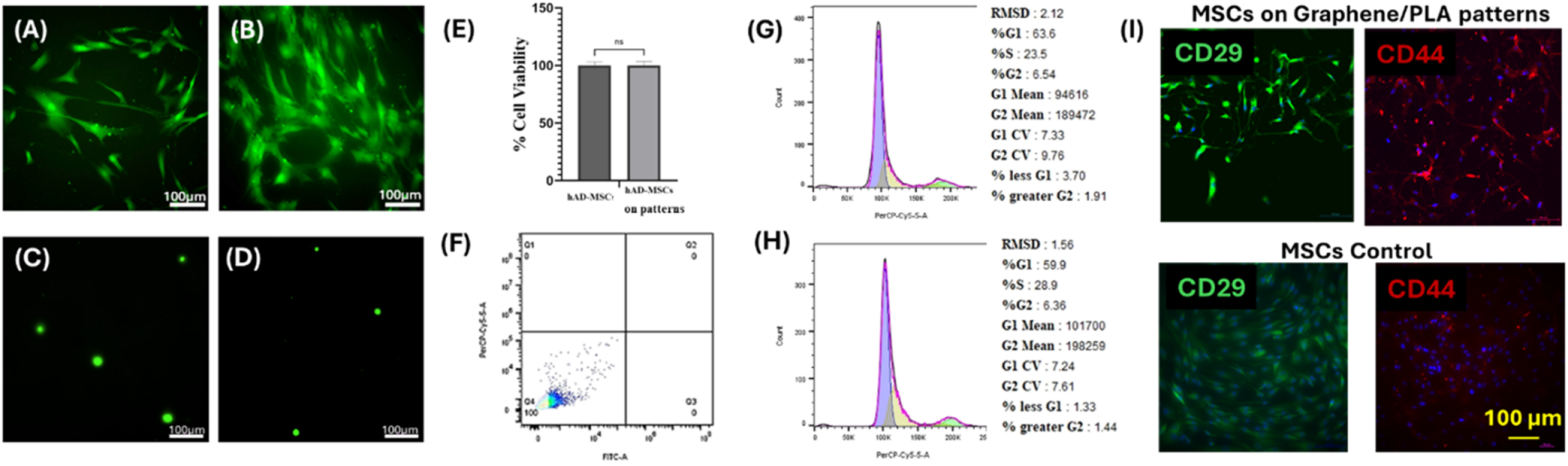
Live/Dead cell assay analysis at 7 days on (A) Control hAD-MSCs and (B) hAD-MSCs on patterned graphene/PLA films. DNA damage analysis using a comet assay at 7 days on (C) Control hAD-MSCs and (D) hAD-MSCs on patterned graphene/PLA films. (E) Cell viability analysis of hAD-MSCs on patterned graphene/PLA films and Control at 7 days. (F) Apoptosis analysis on hAD-MSCs on patterned graphene/PLA films at 7 days. Cell cycle analysis on (G) Control hAD-MSCs and (H) hAD-MSCs on patterned graphene/PLA films at 7 days. (I) ICC analysis of stem cell markers CD29 and CD44 for hAD-MSCs growing on patterned graphene/PLA films and Petri dishes (control) at 7 days.

To demonstrate the proof of concept for the functionality of the devices fabricated using the developed method, two distinct modes of application were selected: (1) a graphene coil-integrated electrode cuff and (2) an IDE graphene cuff-integrated piezoelectric device (Figure 8A,B).

**Figure 8:**
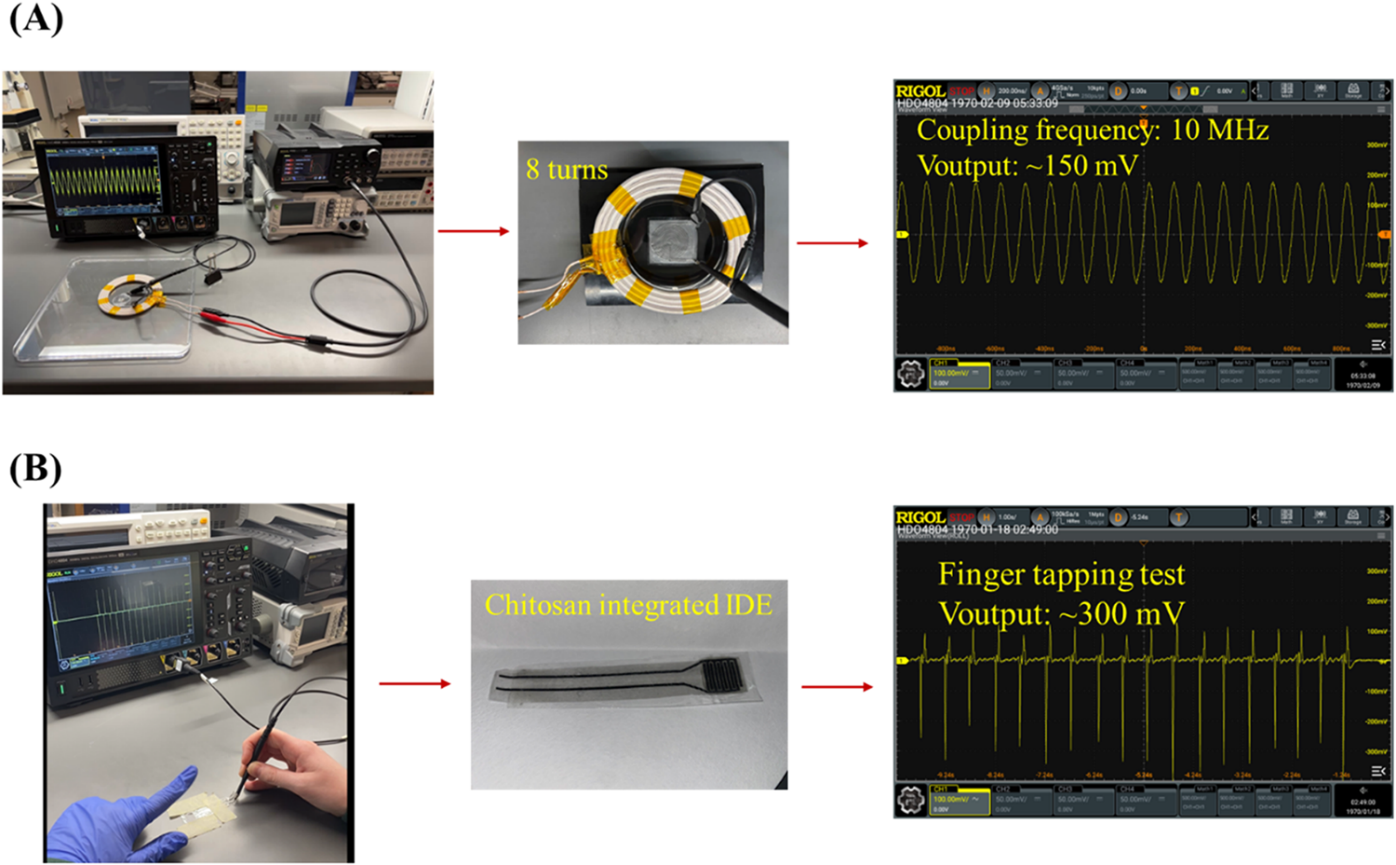
Functional flexible electronic devices fabricated by combining laser cutting with water immersion-based large-area graphene transfer method: (A) graphene coil-integrated electrode cuff and (B) IDE graphene cuff-integrated piezoelectric device.

Wireless electrical stimulation has been used to promote cell and tissue regeneration.^60,61^ In general, to effectively stimulate neural cells and tissues, an output voltage of 50–500 mV is required to provide regeneration, repair, and functional recovery.^62–64^ To mimic the stimulation conditions mentioned in the literature, induction coupling at 10 MHz with a primary antenna and a graphene coil, connected with two nodes for oscilloscope measurements, displayed an output voltage of 150 mV with an input voltage of 10 V from a function generator connected to the primary antenna (Figure 8A). This initial wireless device design, fabricated using our method, demonstrates that the devices we developed can achieve the desired regenerative output voltage ranges.

Similarly, piezoelectric materials are commonly used as energy-harvesting devices, owing to their unique ability to convert mechanical forces into electrical charge.^65,66^ This property has also been used to develop implantable piezoelectric devices that enable voltage ranges capable of producing regenerative effects upon mechanical stimulation.^67–69^ The fabricated device enabled an output voltage of ∼300 mV upon constant finger tapping (Figure 8B). Therefore, these performances provide electrical stimulation conditions that can activate cells and tissues and hold the promise of integration into a range of more specific electrical applications while maintaining integrity and, notably, high conductivity via a biodegradable platform.

In addition to stability, in vitro cytocompatibility, and electrical functionality, it is important to ensure that the developed devices are easy to handle and suture for efficient implantation. For this purpose, the graphene/PLA IDE was integrated with piezoelectric chitosan films of different dimensions and geometries suitable for implantation (Figure 9A). We used a female Sprague–Dawley rat cadaver to test the implantation feasibility at the pudendal nerve for application to treat urinary incontinence. Access to the pudendal nerve was made through the ischiorectal fossa as described previously.^27,70^ It was noted that the developed device was easy to handle and sufficiently flexible to be bent and guided through the tissues. Suture (5–0 Perma-Hand Silk nonabsorbable suture and P-3 reverse cutting needle) was successfully performed to stabilize the device, as seen in Figure 9B,C.

**Figure 9:**
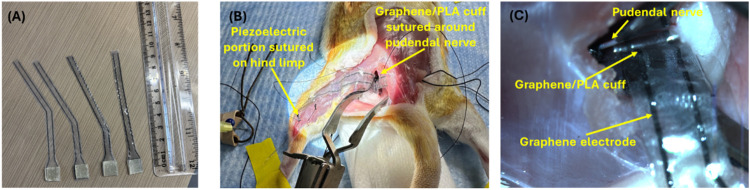
(A) Functional IDE integrated piezoelectric device designed at specific dimensions and shapes for feasible implantation to the stress urinary incontinence cadaver animal model. (B) Feasibility, easy handling, and suturing of the developed device for its implantation in a stress urinary incontinence cadaver animal model. (C) A closer look at the suturing and wrapping of the graphene/PLA cuff around the pudendal nerve.

## 4 Conclusions

This study demonstrated the feasibility of the developed large-area graphene transfer method for fabricating biodegradable, implantable devices for tissue regeneration applications. This post-processing-free, room-temperature-operated graphene transfer method, relying on simple polymer casting and water immersion, enabled the fabrication of conductive graphene-based flexible electronic films, patterns, or devices using biodegradable polymers. Combining this approach with accurate, precise laser cutting also enabled high-throughput fabrication. The patterned graphene/PLA films fabricated by this method demonstrated facile, large-area graphene transfer with an efficiency of ∼100%. The transferred graphene demonstrated structural and electrical integrity and stability. It was also noted that low-temperature treatment reduced surface roughness and increased graphene hydrophobicity, thereby facilitating graphene transfer. The patterned graphene/PLA films exhibited long-term stability in in vitro and in vivo mimicking environments with respect to structural integrity and electrical conductivity. The material-cell interaction studies showed that patterned graphene/PLA films are cytocompatible with various cell types, suggesting the potential for in vivo biocompatibility and implantation. The fabricated graphene coil and IDE-based devices also demonstrated a proof of concept for functionality by producing output voltages within the regenerative potential range. Moreover, devices fabricated using this method were easy to handle and suture during implantation procedures performed on cadaver animals. In conclusion, this method can be used to design and fabricate graphene-based flexible electronic films, patterns, or devices not only to stimulate cells and tissues for regeneration purposes but also for other biomedical applications, such as sensors. Future studies will focus on the design, characterization, and testing of functional, flexible electronic devices and their therapeutic stimulation performance in in vitro and in vivo settings.

## Supplementary Material



## Notes

Viscosity versus shear rate of the graphene ink; load–displacement plot measured during peel test of films under dry and wet conditions; untreated and temperature-treated graphene sheet lift-off pictures using different polymers after water immersion; graphene and PLA layer thickness obtained by stylus profilometer; contact angle of untreated and temperature-treated graphene sheets on glass substrate; *I*–*V* curve of untreated and temperature-treated graphene sheets on glass and PLA substrates, tensile testing of fresh and stored samples; and XPS and SEM results after degradation tests (PDF)

## References

[1] Kim M. S., Almuslem A. S., Babatain W., Bahabry R. R., Das U. K., El-Atab N., Ghoneim M., Hussain A. M., Kutbee A. T., Nassar J., Qaiser N., Rojas J. P., Shaikh S. F., Sevilla G. A. T., Hussain M. M. (2024). Beyond Flexible: Unveiling the Next
Era of Flexible Electronic Systems. Adv. Mater..

[2] Cheng, I.-C. ; Wagner, S. Overview of Flexible Electronics Technology. In Flexible Electronics: Materials and Applications; Wong, W. S. ; Salleo, A. , Eds.; Springer US:: Boston, MA, 2009; pp 1–28.

[3] Jang H., Park Y. J., Chen X., Das T., Kim M.-S., Ahn J.-H. (2016). Graphene-Based Flexible and Stretchable
Electronics. Adv. Mater..

[4] Chen M., Haddon R. C., Yan R., Bekyarova E. (2017). Advances in
Transferring Chemical Vapour Deposition Graphene: A Review. Mater. Horiz..

[5] Chen S., Jiang K., Lou Z., Chen D., Shen G. (2018). Recent Developments
in Graphene-Based Tactile Sensors and E-Skins. Adv. Mater. Technol..

[6] Yong K., Ashraf A., Kang P., Nam S. (2016). Rapid Stencil Mask
Fabrication Enabled One-Step Polymer-Free Graphene Patterning and
Direct Transfer for Flexible Graphene Devices. Sci. Rep..

[7] Ma Y., Zhi L. (2019). Graphene-Based Transparent Conductive Films: Material Systems, Preparation
and Applications. Small Methods.

[8] Langston X., Whitener K. E. (2021). Graphene Transfer:
A Physical Perspective. Nanomaterials.

[9] Ullah S., Yang X., Ta H. Q., Hasan M., Bachmatiuk A., Tokarska K., Trzebicka B., Fu L., Rummeli M. H. (2021). Graphene
Transfer Methods: A Review. Nano Res..

[10] Shivayogimath A., Whelan P. R., Mackenzie D. M. A., Luo B., Huang D., Luo D., Wang M., Gammelgaard L., Shi H., Ruoff R. S., Bøggild P., Booth T. J. (2019). Do-It-Yourself Transfer of Large-Area
Graphene Using an Office Laminator and Water. Chem. Mater..

[11] Zhang X., Xu C., Zou Z., Wu Z., Yin S., Zhang Z., Liu J., Xia Y., Lin C.-T., Zhao P., Wang H. (2020). A Scalable
Polymer-Free Method for Transferring Graphene onto Arbitrary Surfaces. Carbon.

[12] Wu R., Gan L., Ou X., Zhang Q., Luo Z. (2016). Detaching
Graphene
from Copper Substrate by Oxidation-Assisted Water Intercalation. Carbon.

[13] Zhao Y., Song Y., Hu Z., Wang W., Chang Z., Zhang Y., Lu Q., Wu H., Liao J., Zou W., Gao X., Jia K., Zhuo L., Hu J., Xie Q., Zhang R., Wang X., Sun L., Li F., Zheng L., Wang M., Yang J., Mao B., Fang T., Wang F., Zhong H., Liu W., Yan R., Yin J., Zhang Y., Wei Y., Peng H., Lin L., Liu Z. (2022). Large-Area Transfer of Two-Dimensional Materials Free
of Cracks, Contamination and Wrinkles via Controllable Conformal Contact. Nat. Commun..

[14] Hu Z., Li F., Wu H., Liao J., Wang Q., Chen G., Shi Z., Zhu Y., Bu S., Zhao Y., Shang M., Lu Q., Jia K., Xie Q., Wang G., Zhang X., Zhu Y., Wu H., Peng H., Lin L., Liu Z. (2023). Rapid and
Scalable Transfer of Large-Area Graphene Wafers. Adv. Mater..

[15] Li D., Lai W.-Y., Zhang Y.-Z., Huang W. (2018). Printable Transparent
Conductive Films for Flexible Electronics. Adv.
Mater..

[16] Chen Y., Gong X.-L., Gai J.-G. (2016). Progress and Challenges in Transfer
of Large-Area Graphene Films. Adv. Sci..

[17] Wang L., Yu J., Zhang Y., Yang H., Miao L., Song Y. (2017). Simple and
Large-Scale Strategy to Prepare Flexible Graphene Tape Electrode. ACS Appl. Mater. Interfaces.

[18] Jeong S., Jung M.-W., Lee J.-Y., Kim H., Lim J., An K.-S., Choi Y., Lee S. S. (2013). Graphene
Electrodes
Transfer-Printed with a Surface Energy-Mediated Wet PDMS Stamp: Impact
of Au Doped-Graphene for High Performance Soluble Oxide Thin-Film
Transistors. J. Mater. Chem. C.

[19] Hong J.-Y., Jang J. (2012). Micropatterning of
Graphene Sheets: Recent Advances in Techniques
and Applications. J. Mater. Chem..

[20] Kang K., Cho Y., Yu K. J. (2018). Novel Nano-Materials
and Nano-Fabrication Techniques
for Flexible Electronic Systems. Micromachines.

[21] Gao W., Emaminejad S., Nyein H. Y. Y., Challa S., Chen K., Peck A., Fahad H. M., Ota H., Shiraki H., Kiriya D., Lien D.-H., Brooks G. A., Davis R. W., Javey A. (2016). Fully Integrated
Wearable Sensor Arrays for Multiplexed in Situ Perspiration
Analysis. Nature.

[22] Brain-Computer Interfaces: Applying Our Minds to Human-Computer Interaction; Tan, D. S. ; Nijholt, A. , Eds.; Springer London:: London, 2010.

[23] Viventi J., Kim D.-H., Moss J. D., Kim Y.-S., Blanco J. A., Annetta N., Hicks A., Xiao J., Huang Y., Callans D. J., Rogers J. A., Litt B. (2010). A Conformal, Bio-Interfaced
Class of Silicon Electronics for Mapping Cardiac Electrophysiology. Sci. Transl. Med..

[24] Simsar E. G., Cheng P., Dogruel T., Donta M., Jung J., Asante N. A., Sakaguchi D. S., Mallapragada S. K., Kidambi P. R., Uz M. (2025). Few-Layered Conductive
Graphene Foams
for Electrical Transdifferentiation of Mesenchymal Stem Cells Into
Schwann Cell-Like Phenotypes. Adv. Healthcare
Mater..

[25] Hosseini E. S., Manjakkal L., Shakthivel D., Dahiya R. (2020). Glycine–Chitosan-Based
Flexible Biodegradable Piezoelectric Pressure Sensor. ACS Appl. Mater. Interfaces.

[26] Pan H. Q., Kerns J. M., Lin D. L., Liu S., Esparza N., Damaser M. S. (2007). Increased Duration of Simulated Childbirth
Injuries
Results in Increased Time to Recovery. Am. J.
Physiol. Regul. Integr. Comp. Physiol..

[27] Damaser M. S., Broxton-King C., Ferguson C., Kim F. J., Kerns J. M. (2003). Functional
and Neuroanatomical Effects of Vaginal Distention and Pudendal Nerve
Crush in the Female Rat. J. Urol..

[28] Al
Shboul A., Ketabi M., Skaf D., Nyayachavadi A., Yu T. L. F., Rautureau T., Rondeau-Gagné S., Izquierdo R. (2023). Graphene Inks Printed by Aerosol Jet for Sensing Applications:
The Role of Dispersant on the Inks’ Formulation and Performance. Sensors.

[29] Tran T. S., Dutta N. K., Choudhury N. R. (2019). Graphene-Based Inks for Printing
of Planar Micro-Supercapacitors: A Review. Materials.

[30] Ahmed M. R., Mirihanage W., Potluri P., Fernando A. (2023). Highly Stable Graphene
Inks Based on Organic Binary Solvents. Part.
Part. Syst. Charact..

[31] Fakhari A., Fernandes C., Galindo-Rosales F. J. (2022). Mapping the Volume Transfer of Graphene-Based
Inks with the Gravure Printing Process: Influence of Rheology and
Printing Parameters. Materials.

[32] Uz M., Lentner M. T., Jackson K., Donta M. S., Jung J., Hondred J., Mach E., Claussen J., Mallapragada S. K. (2020). Fabrication
of Two-Dimensional and Three-Dimensional High-Resolution Binder-Free
Graphene Circuits Using a Microfluidic Approach for Sensor Applications. ACS Appl. Mater. Interfaces.

[33] Uz M., Jackson K., Donta M. S., Jung J., Lentner M. T., Hondred J. A., Claussen J. C., Mallapragada S. K. (2019). Fabrication
of High-Resolution Graphene-Based Flexible Electronics via Polymer
Casting. Sci. Rep..

[34] Kamyshny A., Magdassi S. (2019). Conductive Nanomaterials
for 2D and 3D Printed Flexible
Electronics. Chem. Soc. Rev..

[35] Oren S., Ceylan H., Schnable P. S., Dong L. (2017). High-Resolution Patterning
and Transferring of Graphene-Based Nanomaterials onto Tape toward
Roll-to-Roll Production of Tape-Based Wearable Sensors. Adv. Mater. Technol..

[36] Vlček J., Lapčík L., Havrdová M., Poláková K., Lapčíková B., Opletal T., Froning J. P., Otyepka M. (2019). Flow Induced HeLa Cell
Detachment Kinetics Show That
Oxygen-Containing Functional Groups in Graphene Oxide Are Potent Cell
Adhesion Enhancers. Nanoscale.

[37] Kozbial A., Li Z., Conaway C., McGinley R., Dhingra S., Vahdat V., Zhou F., D’Urso B., Liu H., Li L. (2014). Study on the
Surface Energy of Graphene by Contact Angle Measurements. Langmuir.

[38] van
Engers C. D., Cousens N. E. A., Babenko V., Britton J., Zappone B., Grobert N., Perkin S. (2017). Direct Measurement
of the Surface Energy of Graphene. Nano Lett..

[39] Rohman N., Mohiuddin T., Al-Ruqeishi M. S. (2023). Surface Free Energy of Graphene-Based
Coatings and Its Component Elements. Inorg.
Chem. Commun..

[40] Weng S.-C., Fuh A. Y.-G., Tang F.-C., Cheng K. T. (2016). Effect
of Surface
Condition on Liquid Crystal Photoalignment by Light-Induced Azo Dye
Adsorption Phenomena. Liq. Cryst..

[41] Yousefzade O., Jeddi J., Vazirinasab E., Garmabi H. (2019). Poly­(Lactic Acid) Phase
Transitions in the Presence of Nano Calcium Carbonate: Opposing Effect
of Nanofiller on Static and Dynamic Measurements. J. Thermoplast. Compos. Mater..

[42] Wang S., Zhang Y., Abidi N., Cabrales L. (2009). Wettability and Surface
Free Energy of Graphene Films. Langmuir.

[43] Chibowski E., Hołysz L., Kip G. A. M., van Silfhout A., Busscher H. J. (1989). Surface Free Energy
Components of Glass from Ellipsometry
and Zeta Potential Measurements. J. Colloid
Interface Sci..

[44] Kajornprai T., Jarapanyacheep R., Saikaeo J., Pojprapai S., Jarukumjorn K., Trongsatitkul T. (2024). Double Percolation of Poly­(Lactic
Acid)/Low-Density Polyethylene/Carbon Nanotube (PLA/LDPE/CNT) Composites
for Force-Sensor Application: Impact of Preferential Localization
and Mixing Sequence. Polymers.

[45] Jung W., Yoon T., Choi J., Kim S., Kim Y. H., Kim T.-S., Han C.-S. (2014). Superstrong Encapsulated
Monolayer
Graphene by the Modified Anodic Bonding. Nanoscale.

[46] Pawar K., Dixit P. (2025). Synergistic Enhancement
of Electroless Film Adhesion on Glass via
Graphene-Oxide Functionalization and Surface Roughening. Mater. Lett..

[47] Maddumage D. C., Panamaldeniya S. A., Kimbulapitiya K. M. M.
D. K., Jayakantha D. N. P. R., Munasinghe M. A. H. M., Pemasiri B. M. K., Gunawardhana N., Dassanayake B. S. (2021). Effect of Annealing Temperature on Electrical Properties
of RGO Thin Films Deposited by Atomized Spray Pyrolysis. Diamond Relat. Mater..

[48] Singh P. K., Singh P. K., Sharma K. (2022). Electrochemical Synthesis
and Characterization
of Thermally Reduced Graphene Oxide: Influence of Thermal Annealing
on Microstructural Features. Mater. Today Commun..

[49] Feng H., Cheng R., Zhao X., Duan X., Li J. (2013). A Low-Temperature
Method to Produce Highly Reduced Graphene Oxide. Nat. Commun..

[50] Lee S.-Y., Lee J.-H., Kim Y.-H., Mahajan R. L., Park S.-J. (2022). Surface
Energetics of Graphene Oxide and Reduced Graphene Oxide Determined
by Inverse Gas Chromatographic Technique at Infinite Dilution at Room
Temperature. J. Colloid Interface Sci..

[51] Wang H., Wang A., Yin H., Ding Y., Li C. (2024). Convenient
Preparation of Expanded Graphite and Graphite Nanosheets as Well as
Improvement of Electrical Conductivity of Polyurethane by Filling
Graphite Nanosheets. Mater. Sci. Eng., B.

[52] Harper A. F., Diemer P. J., Jurchescu O. D. (2019). Contact
Patterning by Laser Printing
for Flexible Electronics on Paper. npj Flexible
Electron..

[53] Liu Z., Shi T., Tang Z., Sun B., Liao G. (2016). Using a Low-Temperature
Carbon Electrode for Preparing Hole-Conductor-Free Perovskite Heterojunction
Solar Cells under High Relative Humidity. Nanoscale.

[54] Mukherjee S. P., Gliga A. R., Lazzaretto B., Brandner B., Fielden M., Vogt C., Newman L., Rodrigues A. F., Shao W., Fournier P. M., Toprak M. S., Star A., Kostarelos K., Bhattacharya K., Fadeel B. (2018). Graphene Oxide Is Degraded
by Neutrophils and the Degradation Products Are Non-Genotoxic. Nanoscale.

[55] Kurapati R., Martìn C., Palermo V., Nishina Y., Bianco A. (2021). Biodegradation
of Graphene Materials Catalyzed by Human Eosinophil Peroxidase. Faraday Discuss..

[56] Kurapati R., Mukherjee S. P., Martín C., Bepete G., Vázquez E., Pénicaud A., Fadeel B., Bianco A. (2018). Degradation of Single-Layer
and Few-Layer Graphene by Neutrophil Myeloperoxidase. Angew. Chem., Int. Ed..

[57] Yadav S., Singh Raman A. P., Raman A. P. S., Meena H., Goswami A. G., Kumar V. B., Jain P., Kumar G., Sagar M., Rana D. K., Bahadur I., Singh P. (2022). An Update on Graphene
Oxide: Applications and Toxicity. ACS Omega.

[58] Ghulam A. N., dos Santos O. A. L., Hazeem L., Pizzorno Backx B., Bououdina M., Bellucci S. (2022). Graphene Oxide (GO) Materials—Applications
and Toxicity on Living Organisms and Environment. J. Funct. Biomater..

[59] Ou L., Song B., Liang H., Liu J., Feng X., Deng B., Sun T., Shao L. (2016). Toxicity of Graphene-Family
Nanoparticles: A General Review of the Origins and Mechanisms. Part. Fibre Toxicol..

[60] Guo H., D’Andrea D., Zhao J., Xu Y., Qiao Z., Janes L. E., Murthy N. K., Li R., Xie Z., Song Z., Meda R., Koo J., Bai W., Choi Y. S., Jordan S. W., Huang Y., Franz C. K., Rogers J. A. (2021). Advanced
Materials in Wireless, Implantable Electrical
Stimulators That Offer Rapid Rates of Bioresorption for Peripheral
Axon Regeneration. Adv. Funct. Mater..

[61] Koo J., MacEwan M. R., Kang S.-K., Won S. M., Stephen M., Gamble P., Xie Z., Yan Y., Chen Y.-Y., Shin J., Birenbaum N., Chung S., Kim S. B., Khalifeh J., Harburg D. V., Bean K., Paskett M., Kim J., Zohny Z. S., Lee S. M., Zhang R., Luo K., Ji B., Banks A., Lee H. M., Huang Y., Ray W. Z., Rogers J. A. (2018). Wireless Bioresorbable Electronic System Enables Sustained
Nonpharmacological Neuroregenerative Therapy. Nat. Med..

[62] Kim H.-S., Baby T., Lee J.-H., Shin U. S., Kim H.-W. (2024). Biomaterials-Enabled
Electrical Stimulation for Tissue Healing and Regeneration. Med-X.

[63] Juckett L., Saffari T. M., Ormseth B., Senger J.-L., Moore A. M. (2022). The Effect
of Electrical Stimulation on Nerve Regeneration Following Peripheral
Nerve Injury. Biomolecules.

[64] ElAbd R., Alabdulkarim A., AlSabah S., Hazan J., Alhalabi B., Thibaudeau S. (2022). Role of Electrical
Stimulation in Peripheral Nerve
Regeneration: A Systematic Review. Plast. Reconstr.
Surg. Global Open.

[65] Jin F., Li T., Yuan T., Du L., Lai C., Wu Q., Zhao Y., Sun F., Gu L., Wang T., Feng Z.-Q. (2021). Physiologically Self-Regulated, Fully Implantable,
Battery-Free System for Peripheral Nerve Restoration. Adv. Mater..

[66] Mao R., Yu B., Cui J., Wang Z., Huang X., Yu H., Lin K., Shen S. G. F. (2022). Piezoelectric Stimulation from Electrospun Composite
Nanofibers for Rapid Peripheral Nerve Regeneration. Nano Energy.

[67] Deng X., Zhou Q., Shi R., Zhuang Y., Lin K. (2025). Recent Progress
in Piezoelectric Biomaterials for Peripheral Nerve Regeneration. BME Horiz..

[68] Zaszczynska A., Sajkiewicz P., Gradys A. (2020). Piezoelectric Scaffolds as Smart
Materials for Neural Tissue Engineering. Polymers.

[69] Bian T., Jiang Y., Cao J., Wu W., Zhang L., Yang Y. (2025). Fabrication of Piezoelectric/Conductive Composite Nerve Conduits
for Peripheral Nerve Regeneration. Colloids
Surf., B.

[70] Jiang H.-H., Pan H. Q., Gustilo-Ashby M. A., Gill B., Glaab J., Zaszczurynski P., Damaser M. (2009). Dual Simulated Childbirth Injuries
Result in Slowed Recovery of Pudendal Nerve and Urethral Function. Neurourol. Urodyn..

