# Supplementary material for: Polymer Casting and Water Immersion-Based Large-Area Graphene Transfer for Flexible Electronics Fabrication
Source: ACS Appl Mater Interfaces. 2026 Feb 16;18(7):10963–78. doi: 10.1021/acsami.5c23601 (PMC12954665; doi:10.1021/acsami.5c23601)
Supplement: Supplementary file 1 [file am5c23601_si_001.pdf]

Supporting Information

Polymer Casting and Water Immersion-based Large Area Graphene Transfer for Flexible Electronics Fabrication

Andrea Zuccaro[1], Ekin G. Simsar[1], Naomi Addai Asante[1], Tugce Dogruel[1], Lan Wang[2], Tejasvini Malakalapalli[2], Piran R. Kidambi[3], Hasan Erbil Abaci[4], Margot Damaser[2,5,6] and **Metin Uz[1,5]***

[1]Chemical and Biomedical Engineering, Cleveland State University, 2121 Euclid Avenue, Cleveland, OH 44115, USA

[2]Biomedical Engineering, Lerner Research Institute, Cleveland Clinic, 9620 Carnegie Avenue N Building, Cleveland, OH 44106, USA

[3]Mechanical and Aerospace Engineering, University of Florida, 939 Center Drive Gainesville, FL 32611, USA

[4]Dermatology and Biomedical Engineering, Columbia University, 622 West 168th Street, New York, NY 10032, USA

[5]Advanced Platform Technology Center, Louis Stokes Cleveland VA Medical Center, 10701 East Boulevard, Cleveland, OH 44106, USA

[6]Glickman Urological and Kidney Institute, Cleveland Clinic, 2050 East 96th St Q Building, Cleveland, OH 44106, USA

***Corresponding Author: Metin Uz, PhD**

Assistant Professor

Chemical and Biomedical Engineering

Cleveland State University, Cleveland, OH, USA

m.uz@csuohio.edu

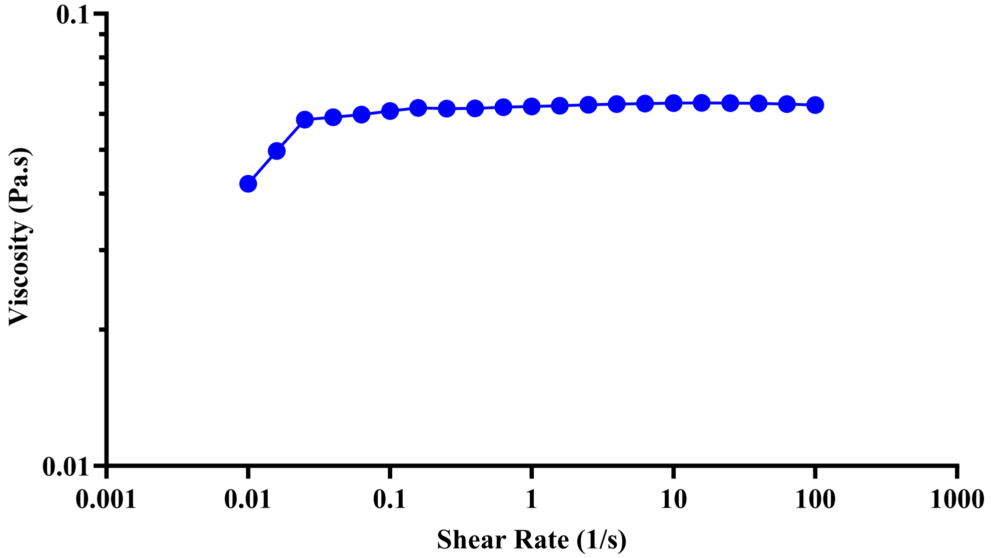

Figure S1. Viscosity versus shear rate of the prepared graphene ink.

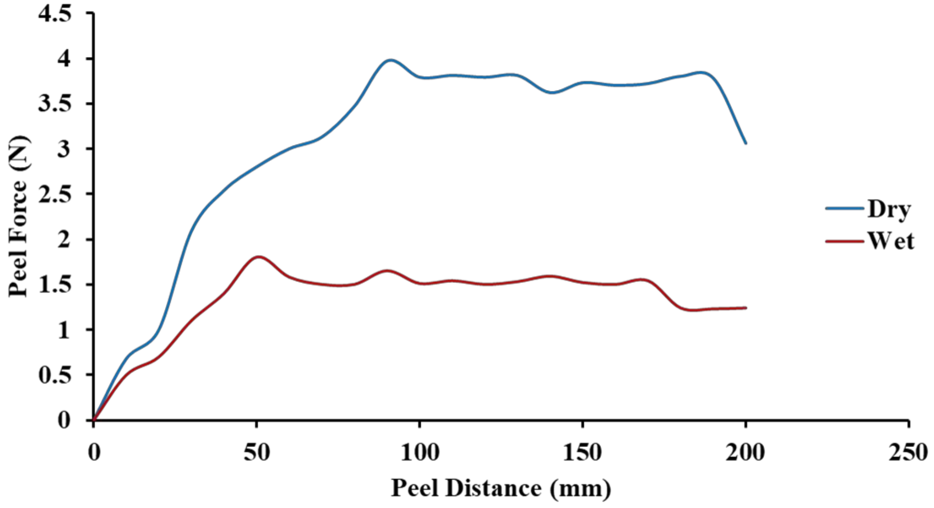

Figure S2. Load–displacement plot measured during peel test for dry and wet conditions.

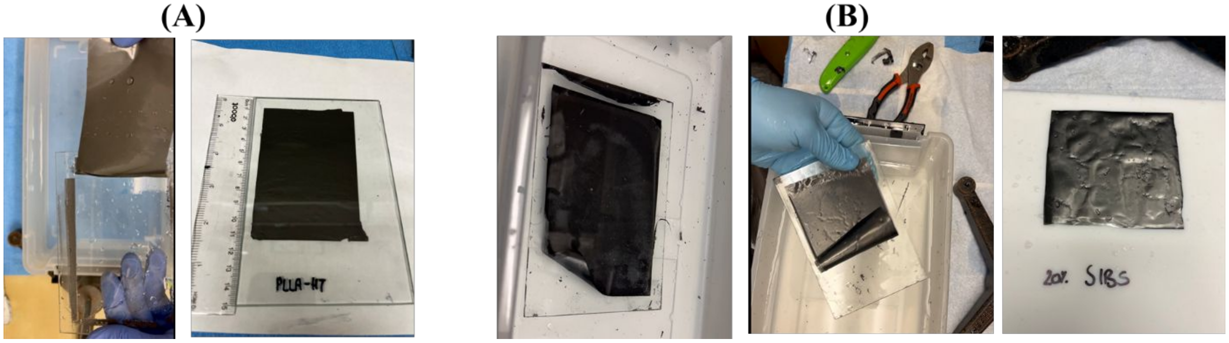

Figure S3. Temperature treated graphene sheet lift off after water immersion obtained by **(A)** PLLA and **(B)** polystyrene-based polymer casting.

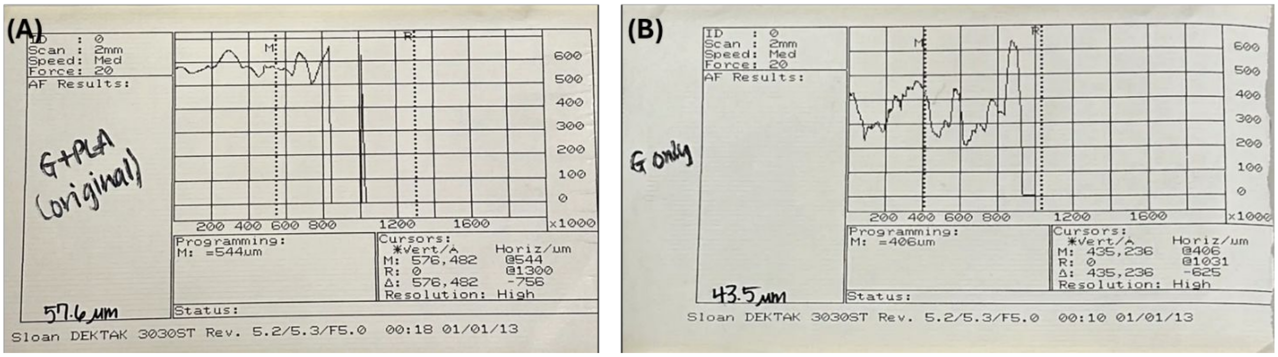

Figure S4. (A) Overall thickness of the graphene/PLA film and **(B)** the thickness of the graphene sheet on the glass substrate.

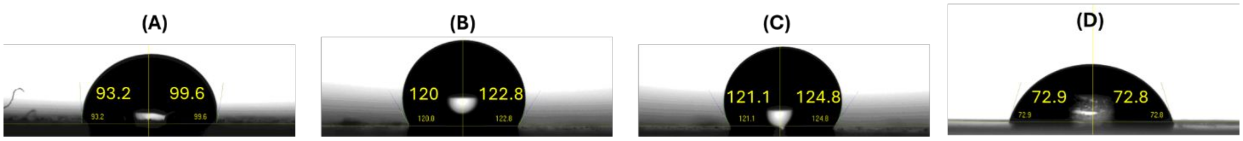

Figure S5. Contact angle of graphene sheet on glass substrate; **(A)** untreated **(B)** temperature treated at 100 °C and **(C)** temperature treated at 150 °C. **(D)** Contact angle on PLA film alone, without graphene transfer.

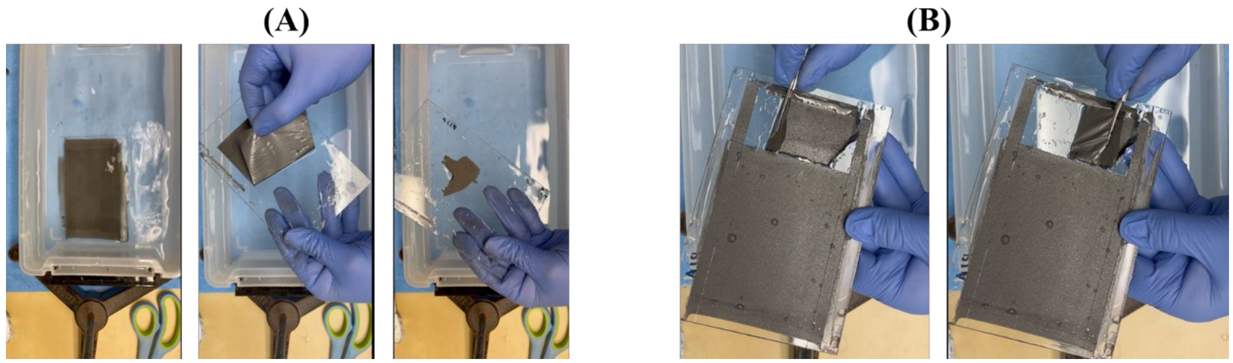

Figure S6. Manual peeling off of untreated graphene sheet from the glass substrate after water immersion obtained by **(A)** PLLA and **(B)** PLA casting.

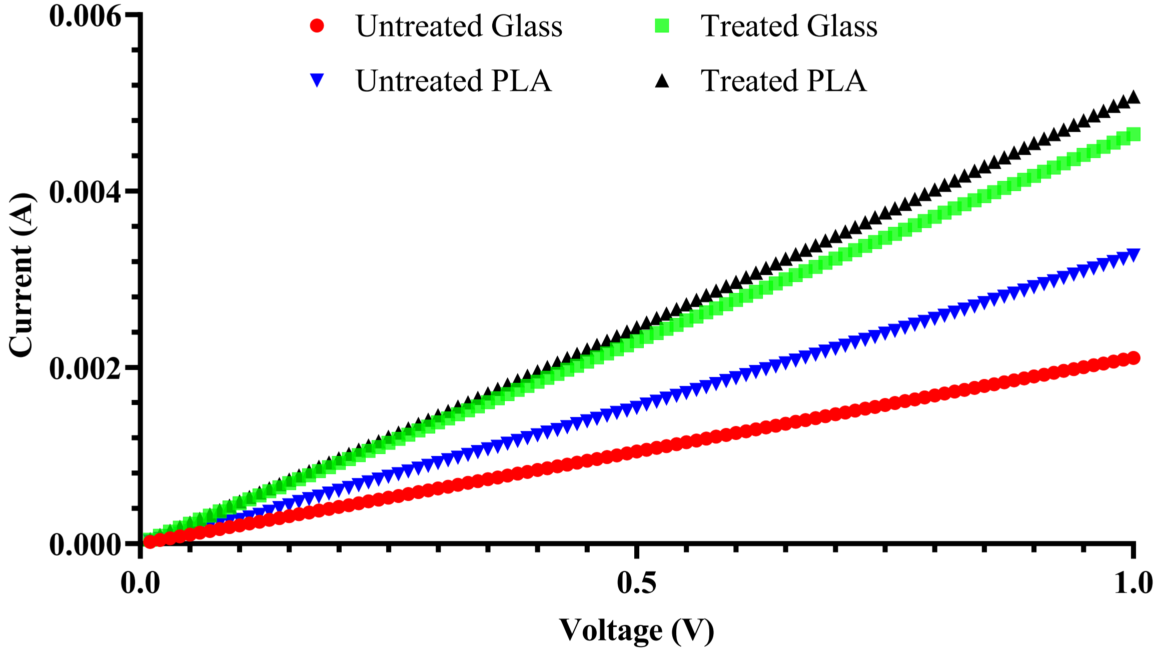

Figure S7. I-V curve of temperature treated and untreated graphene sheets on glass and PLA substrates.

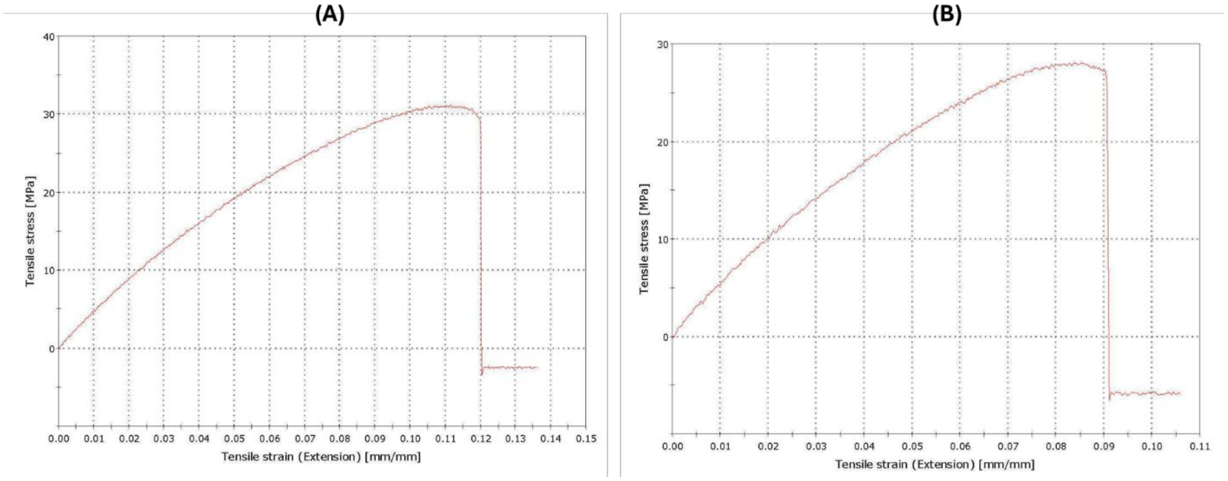

Figure S8. Tensile test results for **(A)** sample stored for 90 days at room temperature and **(B)** fresh sample.

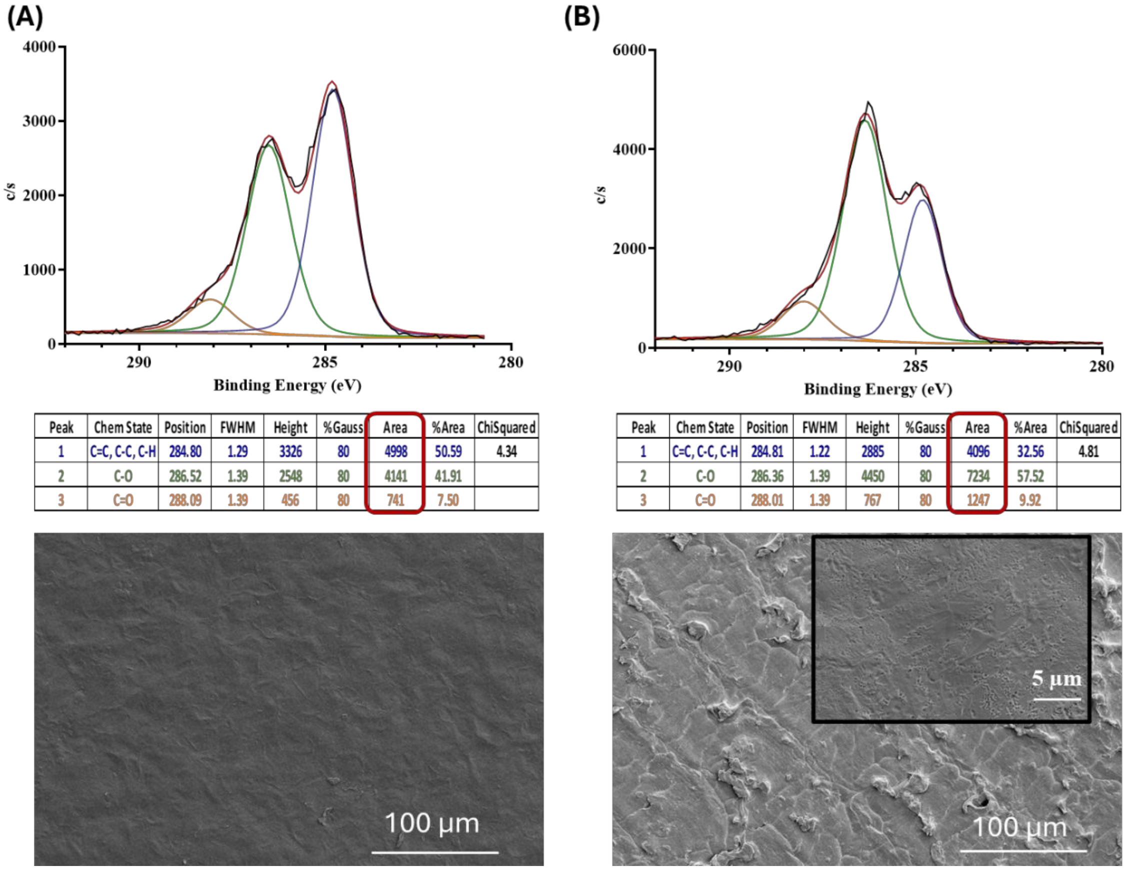

(A)

Peak	Chem State	Position	FWHM	Height	%Gauss	Area	%Area	ChiSquared
1	C=C, C-C, C-H	284.80	1.29	3326	80	4998	50.59	4.34
2	C-O	286.52	1.39	2548	80	4141	41.91	
3	C=O	288.09	1.39	456	80	741	7.50	

(B)

Peak	Chem State	Position	FWHM	Height	%Gauss	Area	%Area	ChiSquared
1	C=C, C-C, C-H	284.81	1.22	2885	80	4096	32.56	4.81
2	C-O	286.36	1.39	4450	80	7234	57.52	
3	C=O	288.01	1.39	767	80	1247	9.92	

Figure S9. XPS analysis and SEM images after **(A)** accelerated degradation and **(B)** enzymatic degradation tests.
